# Preserved Aesthetic Judgements in Parkinson’s Disease: A Case–Control Study Suggests Limited Need for Content Adaptation for Receptive Arts Engagement

**DOI:** 10.3390/jcm15134865

**Published:** 2026-06-23

**Authors:** Blanca T. M. Spee, Domicele Jonauskaite, Bastiaan R. Bloem, Emmy van den Berg, Nina Verhoeven, Dagne Bagdonaviciute, Nicolien Dam, Julia S. Crone, Jorik Nonnekes, David Steyrl, Matthew Pelowski

**Affiliations:** 1 Department of Neurology, Donders Institute for Brain, Cognition and Behavior, Center of Expertise for Parkinson & Movement Disorders, Radboud University Medical Center, 6500 HB Nijmegen, The Netherlands; 2 Department of Cognition, Emotion, and Methods in Psychology, Faculty of Psychology, University of Vienna, 1010 Vienna, Austria; 3 Institute of Psychology, University of Lausanne, 1015 Lausanne, Switzerland; 4 Centre for Cognitive Neuroscience, Department of Psychology, Paris Lodron University Salzburg, 5020 Salzburg, Austria; 5 Department of Rehabilitation, Donders Institute for Brain, Cognition and Behavior, Center of Expertise for Parkinson & Movement Disorders, Radboud University Medical Center, 6500 HB Nijmegen, The Netherlands

**Keywords:** Parkinson’s disease, receptive arts engagement, aesthetic judgments, color-emotion association, case control study

## Abstract

**Background/Objectives:** Parkinson’s disease (PD) is increasingly recognized as a multisystem disorder affecting perceptual, emotional, and reward-related processes. While arts-based interventions in PD have primarily focused on active creative arts engagement, it remains unclear whether receptive arts engagement with visual art—how artworks are perceived and evaluated—is altered. Our objective is to determine whether aesthetic evaluation of visual artworks differs in individuals with PD compared to age-matched healthy controls. We further examine whether emotional interpretation, color-emotion associations, and experiential responses to art viewing are altered. **Methods:** In a cross-sectional case–control study, individuals with PD (*n* = 87) and age-matched healthy controls (*n* = 49) completed two online assessments. Participants evaluated 36 artworks from the Vienna Art Picture System in terms of liking, beauty, and subjective art attributes. Objective image-derived features were computed for each artwork. Interpretable machine learning models were used to test whether evaluation patterns predicted diagnostic group and to identify determinants of aesthetic judgments. Participants further completed a color-emotion association task using ambiguous expressive portraits and reported perceived changes in cognitive, emotional, motivational, and physical states following art viewing. **Results:** Aesthetic evaluation patterns did not support reliable classification of PD status, indicating no systematic group differences in liking, beauty, or attribute-based judgments between PD and controls. Instead, aesthetic judgments were robustly predicted by individual differences and objective artwork properties, including art-historical style, symmetry, complexity, and color-related features, whereas diagnostic group, gender, and age did not contribute to predictions. Emotional interpretation and color-emotion associations were largely comparable between groups, with a single specific deviation in color-emotion mapping. Positive emotions were less frequently associated with pink in people with PD. Self-reported experiential responses to art viewing did not differ significantly between groups. **Conclusions:** Aesthetic evaluation of visual artworks appears largely preserved in people with PD. These findings suggest that, in digital viewing contexts, substantial adaptation of visual content to make it accessible for people with PD may not be necessary, although subtle perceptual and emotional differences may still be relevant. Efforts may instead be better directed toward addressing practical barriers to visual art engagement.

## 1. Introduction

Parkinson’s disease (PD) is traditionally defined by its motor symptoms, yet it is increasingly recognized as a multisystem neurodegenerative disorder affecting perception, emotion, motivation, and reward processing [1]. Beyond tremor and bradykinesia, PD is associated with changes in visual processing, affective evaluation, and cognitive flexibility [2]. These alterations raise important questions about how individuals with PD evaluate complex cultural stimuli, including visual art [3,4].

Engagement with visual art has been associated with emotional well-being and quality of life across clinical and non-clinical populations [5,6]. In PD, arts-based research has primarily focused on active engagement (e.g., art making or creative production), with emerging evidence suggesting benefits for mental health, such as stress reduction [7,8,9,10]. In contrast, receptive arts engagements—how individuals evaluate and experience artworks when viewing them—remain largely unexplored [11]. This distinction is relevant: receptive engagement requires minimal motor involvement and may represent a scalable and accessible form of cultural participation. It is also implicitly assumed that perceptual and affective mechanisms supporting aesthetic evaluation may remain largely intact—a premise that has been only minimally tested in people with PD [12].

Existing findings are mixed. A small-scale study (approximately 20 people with PD compared to 20 age-matched controls) reported no group differences in liking and beauty judgments, although people with PD reported higher emotionality ratings, as an art attribute used to validate personal liking [13,14]. Conversely, another study examining different art styles expressing movement found differences in ratings of beauty, liking, interest, complexity, and visual balance between 43 people with PD and 40 age-matched controls [4].

Aesthetic judgment is not a unitary construct. Contemporary models in art and empirical aesthetics conceptualize it as an integration of perceptual processing, affective response, and subjective value attribution [13]. Beauty and liking judgments are shaped by formal-perceptual features (e.g., complexity, harmony, color properties), content-representational attributes (e.g., emotional expressiveness, symbolism), and individual as well as socio-cultural factors [15,16,17]. Machine-learning approaches have demonstrated that such multidimensional attributes can reliably predict aesthetic judgments across individuals and contexts, suggesting structured patterns in how artworks are appraised [15].

Several features of PD make alterations in aesthetic processing theoretically plausible [3,11]. Visual changes include impairments in color discrimination and contrast sensitivity, alongside differences in emotion recognition and affective processing [18,19,20]. These alterations have been linked to dopaminergic modulation, fronto-striatal dysfunction, and reduced facial expressivity (hypomimia), which may influence embodied simulation during perception [21,22].

Color may also play a particularly important role in this art context [23]. Formal color attributes—either subjectively perceived [15] or objective artwork image-derived features [24] such as lightness, saturation, temperature, and hue variety—significantly influence aesthetic judgments, including liking and beauty [15,17]. At the same time, color carries systematic affective meaning, with relatively consistent color-emotion associations observed across individuals and cultures [25,26,27,28]. In artwork, color contributes to perceived emotional expressiveness and valence [23], and the same is true for colored drawings [29,30]. If PD alters either color perception or emotional processing, the integration of color-based affective cues into aesthetic evaluation may be affected.

Despite this theoretical basis, it remains unclear whether individuals with PD differ systematically in personal preferences (liking) and aesthetic appraisal ratings (beauty), whether such differences are detectable in predictable rating patterns, or whether color-emotion associations are altered when interpreting ambiguous expressive stimuli.

The present study addressed these questions in a cross-sectional case–control design comparing individuals with PD to age-matched controls. We examined three complementary components of receptive arts engagement: First, we investigated aesthetic evaluation of genuine artworks, including liking, beauty, and a comprehensive set of subjective art attribute ratings [15], together with objective image-derived features [24]. Using predictive modeling, we first tested whether participants’ evaluation patterns (liking, beauty, and subjective art attribute ratings) could predict diagnostic group (PD vs. control). We then examined which participant-related and objective artwork image-derived features predicted liking and beauty ratings.

Second, we explored potential group differences in emotional interpretation and color-emotion associations using an artwork portrait (Armand Henrion) task with ambiguous expressive facial stimuli [31]. Third, we examined self-reported experiential changes following art viewing across physical, cognitive, emotional, and motivational domains.

Rather than assuming global deficits in people with PD, the study was guided by the possibility of selective modulation: aesthetic evaluation may or may not remain largely unchanged in people with PD, while specific affective interpretations or experiential dynamics may shift. Clarifying this distinction is clinically relevant. If liking and beauty are largely preserved, cultural accessibility efforts for individuals with PD may not require adaptation of visual content per se. Conversely, if perceptual–affective coupling or emotional experience differs in systematic ways, this could inform the design of museum programs, digital viewing environments including visual cue research [32,33], and arts-based receptive interventions.

## 2. Methods

This study employed a cross-sectional observational case–control design to examine receptive visual arts engagement in individuals with PD compared to age-matched healthy controls. The study combined a hypothesis- and data-driven analysis (part 1) and an exploratory analysis of emotional interpretation, color-emotion associations (part 2), and self-reported experiential changes following online art viewing.

### 2.1. Participants

Inclusion criteria were a clinical diagnosis of Parkinsonism, excluding drug-induced Parkinsonism, and those who had visited a neurology outpatient clinic at one of the four community hospitals. Both people with PD as well as age-matched healthy controls were associated with the ‘Proactive and Integrated Management and Empowerment in Parkinson’s Disease’ (PRIME Parkinson) healthcare innovation project in the South-East region of the Netherlands, which has previously been described in detail elsewhere [34,35].

To describe the study cohort, we collected data on standard demographics, including age, gender identity, country of origin, level of education, living situation, and working status (Table 1). We also collected diagnosis and clinical information on the Hoehn and Yahr scale, global cognition (Montreal Cognitive Assessment (MoCA) short version, maximum 22 points), self-reported perceived health status, and caregiver frequency.

### 2.2. Procedure

Participants with PD were recruited via the outpatient clinic and research platforms associated with the Radboud University Medical Center and the Expertise Centre for Parkinson & Movement Disorders (Nijmegen, The Netherlands). Controls were recruited through the same channels, including partners and caregivers of individuals with PD. All participants participated in the study from January 2025 to April 2025 and provided written informed consent prior to participation.

Participants completed two online questionnaires administered approximately one week apart using the electronic data capture system CASTOR EDC. In the first questionnaire, participants viewed 36 artworks selected from the Vienna Art Picture System (VAPS, [36]), a validated dataset comprising 999 high-quality digital images of genuine artwork (Table S1 in Supplementary Materials for selection of artwork stimuli). The list of aesthetic judgements and subjective as well as objective image-derived art attributes is described hereafter.

Approximately one week later, participants completed the second questionnaire. This included a color-emotion association task based on adapted grayscale self-portraits from the Henrion paradigm (a selection of Armand Henrion self-portraits) [31]), depicting mixed emotional expressions. Five images were presented (see Figures S1–S5 in the Supplementary Materials; permission to present Armand Henrion portraits for non-commercial use received from the Belvedere Museum in Vienna, Austria).

For each image, participants rated perceived emotional valence (negative and positive), control (in control and lack of control), and arousal (calm and activated) using 11-point Likert scales ranging from 0 (“not at all”) to 10 (“very much”). Participants were further asked to select one or multiple perceived emotions from a structured emotion framework called the Geneva Emotion Wheel [37,38], displaying 20 emotion concepts, organized by similarity, and to rate the intensity of each selected emotion concept on the same tool. Finally, participants colored the head bonnets of the Henrion portraits by selecting one of 11 color names (red, orange, yellow, green, turquoise, blue, purple, pink, brown, gray, black). The latter represent all the basic color categories in European languages [39], apart from white, since the bonnet was presented in this color. We additionally included “turquoise” to represent the blue-green section of the color space, as this category is prominent in color-emotion association research [26]. Participants were allowed to use the same color for as many images as they wished.

The second questionnaire additionally assessed the perceived impact of visual art engagement on symptoms across four domains: physical, cognitive, emotional, and motivational. For each domain, participants indicated whether art engagement was associated with a decrease or increase in specific symptoms using a bipolar numerical rating scale ranging from −10 (strong decrease) to +10 (strong increase), with 0 indicating no change. Physical symptoms included rigidity, tremor, dystonia, and bradykinesia. Cognitive symptoms included cognitive stress, cognitive load, focus/concentration, and nervousness. Emotional symptoms included anxiety, sadness, happiness, and peacefulness. Motivational symptoms included feeling energetic, engagement, interest, and apathy.

The study was conducted in accordance with the Declaration of Helsinki. The study protocol was approved by the Medical Ethics Committee Oost-Nederland (METC Oost-Nederland), Radboudumc (CMO file numbers 2021-12985 and 2024-1740).

### 2.3. Artwork Stimuli Selection and Art Attribute Description

The selection of artworks was operationalized based on two objective artwork related features: (1) level of representativeness to abstractness, categorized according to art-historical style classifications (Baroque and Rococo, *n* = 6; Impressionism, *n* = 6; Expressionism *n* = 6; Cubistic tendencies, *n* = 9; and Symbolism, *n* = 9; in addition, artworks were balanced across content categories, with 12 scenes, 12 landscapes, and 12 works toward abstraction, ensuring coverage across the representational–abstract spectrum); (2) quantified image saturation parameters, specifically lightness and chroma.

Three candidate subsets were randomly drawn from the 999 images, counterbalanced along art-historical style classifications. From these, the subset with the most balanced distribution of lightness and chroma values across the representational–abstract spectrum was selected (see results of lightness and chroma values Table S2). Additionally, image content diversity was ensured by including artworks depicting landscapes, scenes, and abstract content (see Table S1).

Artworks were presented digitally on participants’ personal devices at home. In the study information letter, participants were instructed to complete the task on a desktop or tablet device rather than a mobile phone to ensure adequate image display. Image presentation order was first randomized, and participants were randomly assigned to one of two counterbalanced presentation sequences.

**Aesthetic judgments and subjective art attributes:** For each artwork, participants rated their personal preference (“liking”) and aesthetic appraisal (“beauty”) on 11-point Likert scales ranging from 0 (“not at all”) to 10 (“very much”). In addition, participants evaluated each artwork on a comprehensive set of subjective art attributes using 21-point bipolar Likert scales ranging from −10 to +10. The assessed attributes encompassed both content-representational and formal-perceptual dimensions. Content-representational attributes included emotional expressiveness (emotionless to emotionally loaded), valence (negative to positive), imaginativeness (realistic to imaginative), abstractness (representational to abstract), symbolism or ambiguity (unambiguous to ambiguous), and arousal (calming to activating). Formal-perceptual attributes included visual harmony (inharmonious to harmonious), complexity (simple to complex), perceived color temperature (cool to warm), perceived color saturation (soft/pastel to vibrant/intense), perceived color lightness (dark to light), brushstroke quality (loose/rough to controlled/fine), and pictorial depth (two-dimensional to three-dimensional).

**Objective art image attributes:** Objective art image attributes were calculated to capture quantitative visual properties of the artworks. Two basic color statistics were computed directly from the images: mean image lightness (MeanL) and mean chroma (MeanC), derived from the CIELab color space and reflecting the overall luminance and color intensity of the image.

Additional image statistics were obtained using the computational framework described elsewhere [24], which quantifies structural and color distribution properties of images. These variables included mirror symmetry, visual complexity, compositional balance, mean saturation channel (mean.S.channel), and standard deviation of the saturation channel (std.S.channel). Mirror symmetry quantifies the degree of reflectional symmetry in the image; complexity reflects the structural richness of visual elements; and the saturation-channel statistics describe the distribution and variability of color saturation across the image (see [24] for a detailed description of these metrics and their computation).

### 2.4. Study Endpoints

Primary endpoints: (i) The primary analytical objectives concerned aesthetic evaluation and its relation to PD status. In part 1, two primary modeling targets were defined. First, diagnostic group (PD vs. control) served as the dependent variable to test whether aesthetic evaluation patterns (including liking, beauty, and subjective art attribute ratings) allowed reliable inference of PD status. (ii) Second, aesthetic judgments—operationalized as liking ratings (personal preference) and beauty ratings (aesthetic appraisal)—served as dependent variables in regression models examining the contribution of individual differences, demographic characteristics (diagnostic group, gender identity, and age), and objective image-derived features described in the subchapter “Objective art image attributes.” Individual differences were modeled using subject ID, allowing the models to capture participant-specific baseline tendencies in aesthetic ratings across the artworks. In an exploratory analysis, medication usage (levodopa only vs. dopamine agonists with or without levodopa) and disease severity (HY stage) were included as additional predictors in the regression models.

**Secondary endpoints** included emotional interpretation in the Henrion task (dimensional ratings of valence, control, and arousal) operationalized as categorical emotion selections including intensity levels as well as assigned color categories, and self-reported symptom changes following art viewing across physical, cognitive, emotional and motivational domains.

### 2.5. Data Analysis

#### 2.5.1. Interpretable Machine Learning-Based Data Analyses Framework

To examine whether aesthetic evaluation patterns allow inference of diagnostic status and to identify predictors of aesthetic judgments, we implemented an out-of-sample inference, an interpretable machine learning framework following our previous work [40]. Analyses were conducted in Python (v3.12) using scikit-learn (v1.6.1) and lightgbm (4.6.0) library [41,42].

Separate models were trained for three targets: (1) diagnostic group (PD vs. control; classification task), (2) liking ratings, and (3) beauty ratings (both regression tasks).

Model performance and generalizability was evaluated using nested cross-validation (CV) to reduce overfitting and selection bias [43,44]. In the outer CV, the data (5832 rating observations) were split into training (80%) and testing (20%) sets. For classification, a grouped split ensured that all observations from a participant were assigned to either training or testing data.

Model parameters were optimized within an inner (nested) CV using randomized search. The best-performing parameter set was then used to train the model in the outer CV. Performance was evaluated on held-out test data using balanced accuracy (classification) and prediction *R*^2^ (regression), averaged across outer CV iterations [45].

Prediction models were implemented using Gradient Boosted Decision Trees (GBDT), which capture nonlinear relationships and higher-order interactions between predictors while remaining robust to multicollinearity and outliers [42].

Model interpretation was performed using SHAP (SHapley Additive exPlanations) values [46,47], which estimate the marginal contribution of each predictor to the model’s output. Mean absolute SHAP values were used to determine the relative importance of predictors across models. SHAP values describe, for each feature, the average absolute change in the model’s predicted rating when that feature is considered, given the other features and the learned model. Larger SHAP values indicate a larger influence on the model’s output in the units of the prediction (liking or beauty ratings). SHAP summaries provide an interpretable ranking of influence on predictions but should not be interpreted as a decomposition of explained variance across predictor classes. 

Statistical significance of model performances and predictor importances was assessed using trained models on shuffled data, applying a modified paired *t*-test that accounts for sample dependency introduced by cross-validation procedures [48,49]. Bonferroni correction was applied for multiple comparisons where appropriate. Code and data are available in the public repository: https://github.com/univiemops/aesthetic-preferences-in-pd (accessed on 8 June 2026).

All questionnaire items were mandatory; therefore, no item-level missing data were present. Participants who did not complete or submit the questionnaire were excluded prior to analysis.

#### 2.5.2. Statistical Analyses of Secondary Outcomes

Dimensional emotion ratings (valence, control, arousal) and self-reported experiential changes following art viewing were compared between participants with PD and age-matched controls using Welch’s independent-samples *t*-tests. Means, standard deviations, *t*-values, degrees of freedom, two-tailed *p*-values, and Cohen’s *d* effect sizes were reported. 

To examine group differences (PD vs. control) in categorical emotion selections (Geneva Emotion Wheel), color choice, and color-emotion combination, we used Fisher’s Exact Tests. Due to the presence of small, expected cell frequencies (*n* < 5), *p*-values were computed using Monte Carlo simulation with 5000 iterations. Because the contingency tables exceeded a 2 × 2 structure, Odds Ratios (OR) could not be calculated for the omnibus associations. Instead, the strength of the relationship was reported using Cramer’s *V* as an effect size. All tests were two-tailed with a significance threshold of *α* = 0.05. 

#### 2.5.3. Sample Size

The number of participants followed best practices in artwork rating studies, where a sample of at least 10 judges is known to provide stable scoring with good reliability [50,51,52]. However, since precise sample size justifications for complex, high-dimensional, multivariable models from the machine learning field have not yet been standardized, our power analysis adheres to the most recent recommendations [53,54]. Specifically: (1) A minimum of 50 samples is necessary to initiate any meaningful machine learning-based data analysis [41]. (2) Having 10 to 20 samples per degree of freedom (predictor) is considered reasonable, suggesting a total requirement of 170 to 340 ratings (samples) for the present study [55,56]. Furthermore, a power analysis using a two-tailed Student’s *t*-test, with an alpha of 0.05 and a power of 0.8, indicates that 620 ratings (samples) would be necessary to detect small effects of Cohen’s *d* = 0.2 [57]. Similar studies in the field have demonstrated good model fit with over 50 participants [52,58]; considering we have ratings from 36 artworks, we intend to collect a minimum of 1800 samples.

## 3. Results

Demographic and clinical characteristics are presented in Table 1. The final sample was 136 participants. A total of 14 participants were excluded due to incomplete questionnaire data (i.e., no responses at either timepoint). Nine additional participants were excluded from the self-reported experiential changes analysis due to incorrect questionnaire responses.

### 3.1. Machine Learning Analyses of Aesthetic Evaluation

#### Classification of Diagnostic Group Based on Subjective Evaluation Patterns

A supervised classification model was trained to test whether PD status could be predicted from participants’ aesthetic evaluation patterns across artworks. Predictive performance did not exceed chance level (mean accuracy = 0.495 ± 0.027), and classification accuracy did not differ significantly from models trained on shuffled labels (*p* = 0.541).

Model interpretation using SHAP values suggested that attributes such as symbolism and beauty contributed most strongly to the model predictions. Symbolism and beauty showed statistical significance prior to correction for multiple comparisons, but these effects did not remain significant after correction. However, after Bonferroni correction for the 16 predictors included in the model (*α* = 0.0031), only symbolism remained statistically significant in the control class. Given that the overall model performance did not exceed chance level, these feature contributions should be interpreted with caution. All detailed model outputs and feature importance results are available in the public data repository https://github.com/univiemops/aesthetic-preferences-in-pd (accessed on 8 June 2026).

### 3.2. Regression Models Predicting Liking and Beauty

Regression models predicting liking and beauty ratings showed predictive performance substantially above chance level (Table 2). Model performance was significantly higher than models trained on shuffled labels, indicating that aesthetic evaluations followed systematic patterns rather than random variation.

**Figure 1:**
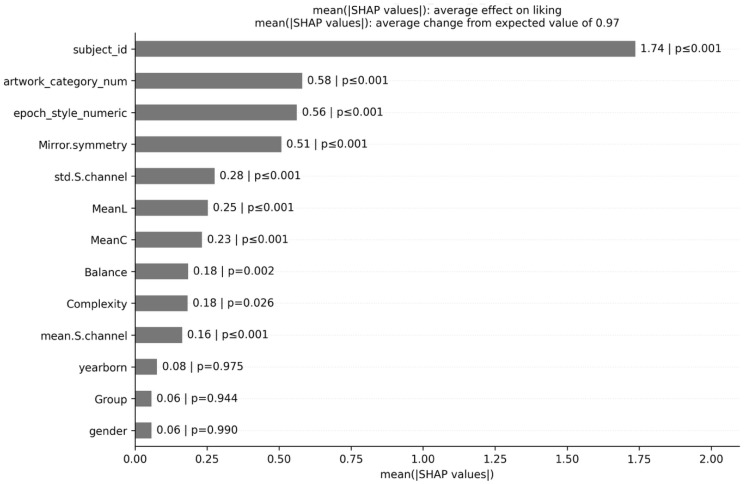
Rank-based predictor importance for the regression model predicting liking ratings. Predictor importance is quantified using mean absolute SHAP values, indicating the average contribution of each predictor to the model’s predictions across cross-validation iterations. Bars are ordered by decreasing importance. Statistical significance of predictor contributions is indicated by *p* < 0.05 and *p* < 0.004 after Bonferroni correction for multiple comparisons.

**Figure 2:**
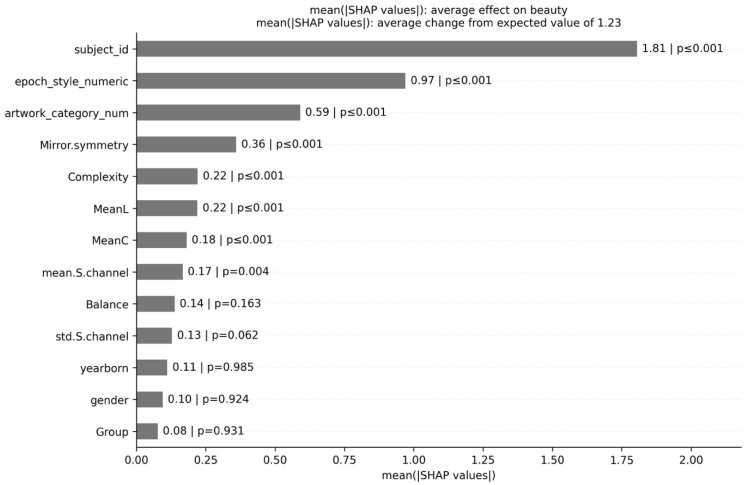
Rank-based predictor importance for the regression model predicting beauty ratings. Predictor importance is quantified using mean absolute SHAP values, indicating the average contribution of each predictor to the model’s predictions across cross-validation iterations. Bars are ordered by decreasing importance. Statistical significance of predictor contributions is indicated by *p* < 0.05 and *p* < 0.004 after Bonferroni correction for multiple comparisons.

Feature importance analyses using SHAP values revealed that subject ID was the strongest predictor in both models, indicating substantial individual differences in aesthetic evaluation (Figure 1 and Figure 2). Beyond these individual differences, several objective art image attributes and artwork-related characteristics significantly contributed to aesthetic judgments.

Regarding art-historical style (epoch style), both liking and beauty ratings showed a negative association with increasing stylistic abstraction. Artworks from Baroque and Rococo and Impressionistic Tendencies were generally evaluated more positively, whereas Expressionistic, Cubistic, and particularly Surrealistic Tendencies were associated with lower aesthetic ratings.

A similar pattern emerged for the artwork category, with landscape paintings receiving higher liking and beauty ratings than scenes or works tending toward abstraction.

Among the objective art image attributes, mirror symmetry, visual complexity, and several color-related image statistics contributed to predictions. Mirror symmetry showed a non-linear association, with ratings decreasing from low to moderate symmetry values and increasing again at higher symmetry levels. Visual complexity contributed more strongly to beauty judgments, with aesthetic evaluations increasing for artworks with higher complexity levels. Color-related attributes—including mean image lightness (MeanL), mean chroma (MeanC), mean saturation (mean.S.channel), and saturation variability (std.S.channel)—also showed systematic associations with aesthetic judgments. For example, mean lightness exhibited an inverted U-shaped relationship, with higher ratings for artworks with moderate luminance values, whereas chroma showed a wave-like pattern across color intensity levels.

Among the individual and demographic characteristics included in the models (group factor, subject ID, gender identity, and age), only subject ID significantly contributed to predictions, whereas diagnostic group (PD vs. control), gender identity, and age did not significantly influence either liking or beauty ratings, even prior to multiple comparison correction.

Medication usage and HY score did not significantly contribute to model predictions and showed low relative importance compared to other predictors (full results are reported at https://github.com/univiemops/aesthetic-preferences-in-pd, accessed on 8 June 2026).

### 3.3. Emotional Interpretation and Color–Emotion Associations

#### 3.3.1. Dimensional Emotion Ratings: Valence, Control, and Arousal

Across the five Henrion portraits (Figures S1–S5 in Supplementary Materials), no consistent group differences were observed in perceived valence or arousal. However, significant differences emerged in perceived emotional control for two images. For Picture 2 (t = 2.236; [0.111, 1.858]; *p* = 0.028) and Picture 3 (t = 2.041; [0.020, 1.493]; *p* = 0.044), control participants rated the depicted expressions as reflecting significantly greater control compared to participants with PD (see for full results Tables S3–S7 in Supplementary Materials). No other significant differences were observed in dimensional ratings.

#### 3.3.2. Categorical Emotion Selection: Geneva Emotion Wheel

A significant group difference was observed for low-control negative emotions in Picture 2. Participants with PD were less than half as likely to select low-control negative emotions compared to controls (*p* = 0.045, Cramer’s *V* = 0.158, see Table S8 and File S1 for full results). No other categorical emotion differences reached significance across images. Based on standardized residuals, participants with PD were less likely to choose low control and negative emotions than control participants (*p* < 0.007), irrespective of color choice (see File S2).

#### 3.3.3. Color Selection

No significant group differences in color choice were observed at the level of individual pictures. However, when collapsing across all stimuli, the distribution of color choices differed significantly between groups (Fisher’s exact test, *p* = 0.026, Cramer’s *V* = 0.179; for full results, see Table S8 in the Supplementary Materials). Based on standardized residuals, participants with PD were less likely to choose pink than control participants (*p* < 0.003), irrespective of perceived emotion. Moreover, there seems to be a trend that participants with PD tend to choose the gray color more often than control participants (*p* = 0.054; for full results, see Tables S9 and S10 in Supplementary Materials).

#### 3.3.4. Color-Emotion Links

**Figure 3:**
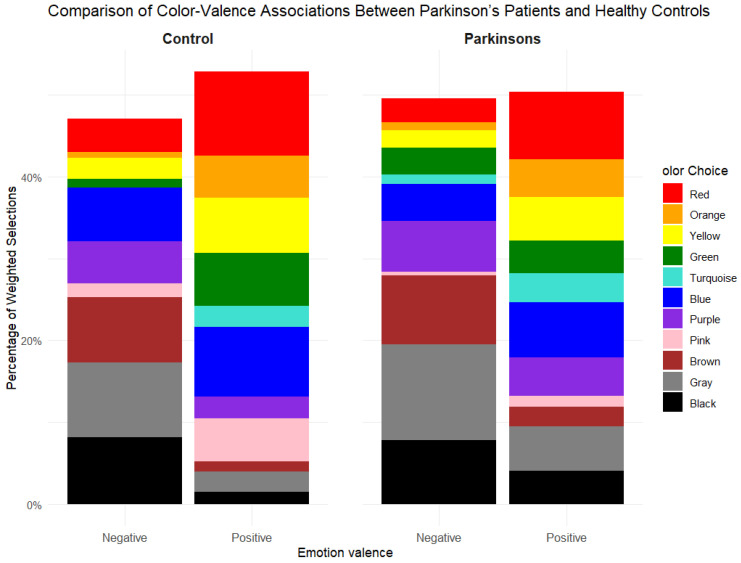
Combined color-emotion choices per category (positive and negative) for self-portraits 1 to 5 of Armand Henrion.

When separating emotions into four categories (i.e., positive-high control, positive-low control, negative-high control, negative-low control) and pooling across the five pictures, no significant group difference emerged across the color categories (Fisher’s exact test, *p* = 0.617, Cramer’s *V* = 0.215; for full results see Table S8 in the Supplementary Materials). When emotions were separated into two categories only (i.e., positive and negative), no significant group difference emerged for positive (Fisher’s exact test, *p* = 0.207, Cramer’s *V* = 0.213) or negative (Fisher’s exact test, *p* = 0.685, Cramer’s *V* = 0.168; see Tables S10 and S11 in the Supplementary Materials and File S3). Based on standardized residuals, participants with PD were less likely to choose pink (*p* < 0.018) than control participants for positive emotions (see Figure 3). More generally, both PD and control participants were more likely to choose orange, turquoise (PD only), green (control only), and red for positive than negative emotions, and brown, gray, and black for negative than positive emotions.

When emotions were separated by control (i.e., high control, low control), irrespective of valence, no group difference emerged for high control (Fisher’s exact test, *p* = 0.708, Cramer’s *V* = 0.133), or for low control (Fisher’s exact test, *p* = 0.816, Cramer’s *V* = 0.127, see Tables S10 and S11 in Supplementary Materials and File S3). Curiously, both PD and control participants were not likely to choose any color for high rather than low control emotions, and vice versa. There were also no significant group differences for individual portraits (see File S3 and Figures S6–S11 in the Supplementary Materials).

### 3.4. Self-Reported Experiential Changes Following Art Viewing

Between-group comparisons revealed no statistically significant differences between participants with PD and controls in the magnitude of reported symptom changes across physical, cognitive, emotional, and motivational domains, when collapsing symptoms to one domain.

Also, when examining increases or decreases in symptoms separately, no significant group differences were observed in cognitive, emotional, or motivational domains (all *p* ≥ 0.415 for increases; see Table S12 in the Supplementary Materials). For decreases in symptoms, no statistically significant differences were detected across domains (all *p* ≥ 0.337; see Table S13 in the Supplementary Materials). Physical symptom changes were reported only by participants with PD and, therefore, were not included in between-group statistical comparisons.

Analyses of absolute symptom change (i.e., magnitude irrespective of direction) likewise revealed no significant group differences in cognitive, emotional, or motivational domains (all *p* ≥ 0.655; see Table S14 in the Supplementary Materials).

### 3.5. Descriptive Patterns of Experiential Changes Following Art Viewing

Although statistical comparisons were non-significant, descriptive visualization separating the symptoms within one domain shows that the most experienced changes reported after viewing art are increases in focus/concentration and curiosity across groups. However, within emotional and cognitive domains, a larger proportion of participants in the PD group reported symptom changes. In particular, people with PD reported increases in cognitive load and nervousness, changes in sadness, and increases in anxiety more frequently than controls, suggesting slightly greater variability in reported changes among PD patients. For full results, see Figures S12–S16 in the Supplementary Materials.

## 4. Discussion

The present study investigated receptive engagement with visual art in individuals with PD across three complementary domains: aesthetic evaluation of artworks, emotional interpretation and color-emotion associations in expressive portrait stimuli, and self-reported experiential changes following art viewing. Several key findings emerged. First, aesthetic evaluation patterns—including liking, beauty, and subjective art attributes—did not allow reliable classification between PD and healthy controls. Second, machine learning models showed that aesthetic judgments of liking and beauty were primarily predicted by individual differences and objective artwork characteristics rather than by diagnostic group or demographic factors. Third, while most emotional and color-related interpretations did not differ between groups, subtle differences emerged in perceived emotional control and aggregated color selections. Finally, self-reported experiential effects of art viewing did not significantly differ between individuals with PD and controls, although descriptive patterns suggested somewhat greater variability in reported emotional and cognitive changes among participants with PD.

### 4.1. Aesthetic Evaluation Appears Largely Preserved in Parkinson’s Disease

The central finding of the present study is that aesthetic judgments of artworks appear largely preserved in individuals with PD. The classification model trained on aesthetic evaluation patterns did not perform above chance level, indicating that the combination of liking, beauty, and subjective art attribute ratings did not contain diagnostic information that could reliably distinguish participants with PD from controls. This finding aligns with previous work reporting comparable liking and beauty judgments between individuals with PD and control participants [11], suggesting that the perceptual–affective mechanisms supporting aesthetic evaluation remain largely intact despite the broader multisystem changes associated with the disorder.

Regression models predicting liking and beauty ratings further support this interpretation. Both models showed robust predictive performance, confirming that aesthetic judgments follow structured and systematic patterns rather than random variation. Importantly, the strongest predictor in both models was subject-ID, highlighting the dominant role of individual differences in aesthetic evaluation [14]. The high SHAP importance of subject-ID indicates that stable individual differences influence predicted ratings more than any single artwork feature, on average. These SHAP summaries should not be interpreted as a decomposition of explained variance across predictor classes. Quantifying variance attributable to predictors would require a separate variance-partitioning analysis, which is beyond the scope of the present work. Beyond subject-ID, aesthetic judgments were shaped by a range of objective artwork properties, including art-historical style, artwork category, visual symmetry, complexity, and color-related image attributes. These findings are consistent with prior research in art and empirical aesthetics, demonstrating that both perceptual image features and viewer-specific factors contribute to aesthetic judgments, considering they share the same socio-cultural environment [15,16,17].

Crucially, the diagnostic group did not significantly contribute to either the liking or beauty models, even before correction for multiple comparisons. Importantly, the absence of classification performance should not be interpreted as evidence of equivalence between groups, but rather as an indication that no systematic group-level differences were detectable within the present study design. This suggests that the basic integration of perceptual and affective cues underlying aesthetic appraisal remains largely unaffected in PD. From a theoretical perspective, this is notable given that PD is associated with alterations in dopaminergic signaling and fronto-striatal circuits involved in reward and valuation processes [2]. The present results, therefore, indicate that, at least in the context of receptive arts engagement, specifically when viewing artworks in the digital environment, these neurobiological changes appear not to translate into measurable differences in personal preference and aesthetic appraisal patterns.

However, we did find some significant results that might be related to socio-cultural training and cohort specificity regarding the age (on average >70) of the whole cohort (people with PD and control): artworks from Baroque and Rococo and Impressionistic traditions were generally evaluated more positively than more abstract styles such as Expressionistic, Cubistic, or Surrealistic tendencies. Similarly, landscape paintings received higher ratings than scenes or more abstract works, reflecting well-documented personal preferences for representational imagery and naturalistic content [5,15]. Objective image properties—including symmetry, complexity, and color statistics—also systematically influenced aesthetic judgments, reinforcing the idea that perceptual structure and visual organization play a central role in shaping aesthetic experiences.

### 4.2. Subtle Differences in Emotional Interpretation and Color–Emotion Associations

Although personal preferences and aesthetic appraisal itself appeared largely preserved, small differences emerged in the interpretation of ambiguous emotional face expressions. Across the two groups, the interpretation of Armand Henrion portrait stimuli went in line with the previous reports [31], but participants with PD rated two Armand Henrion portrait stimuli (Self-portrait 2 and 3 of Armand Henrion; Tables S4 and S5 in the Supplementary Materials) as reflecting lower levels of perceived emotional control compared with control participants. This finding may relate to previously reported alterations in emotion recognition and affective processing in PD. However, these group differences were limited to specific stimuli and did not extend more broadly. Similarly, differences in categorical emotion selections using the Geneva Emotion Wheel were limited to a single portrait (Self-portrait 2 of Armand Henrion; Table S8 in Supplementary Materials), with participants with PD being less likely to select low-control negative emotions than control participants. Taken together, these results suggest that while basic emotional interpretation remains largely comparable between the two groups, subtle shifts in the perception of emotional control may occur when interpreting ambiguous expressive cues.

When it came to color selections, irrespective of perceived emotion and aggregated across all Armand Henrion portraits, participants with PD were less likely to choose pink compared to control participants. Finally, when considering both color and emotion choices, color-emotion associations were largely consistent across groups, with no significant differences in color choices at the level of individual stimuli. When aggregating responses across all portraits, a small overall group difference in color selection emerged. Participants with PD were less likely to select pink for positive portraits compared to control participants, while there were no group differences in color selections for negative emotions. This observation is curious because they avoided a color typically associated with positive emotional valence (i.e., pink) for stimuli perceived as positive, deviating from widely observed color-emotion correspondences [26]. It either means that pink is not a positive color for people with PD or that this color is not very relevant at all, since people with PD generally avoided pink. Such results caution practitioners from using color as an indicator of affective state (e.g., [29,59,60,61]), as not all color-emotion correspondences are equally prevalent in people with and without PD. However, apart from pink, all other color-emotion correspondences followed the expected ones. Namely, orange, green, and turquoise were perceived as positive while black, brown, and gray were perceived as negative colors. Red was also perceived as more positive than negative, going against its generally ambivalent nature (i.e., being associated with both positive and negative emotions [26,62]). Curiously, there was no color choice differentiation in terms of control (i.e., being more often chosen for high vs. low control emotions), neither in participants with PD nor controls, going against explicit color-emotion associations [26,63].

Overall, the origins of why people with PD avoided pink are unknown. It might suggest subtle alterations in the integration of perceptual color cues, or differences in color preferences, especially considering that pink is a highly gendered color and thus typically avoided by men [64,65,66].

### 4.3. Experiential Responses to Art Viewing

The final component of the study examined self-reported experiential changes following art viewing. Across physical, cognitive, emotional, and motivational domains, no statistically significant differences were observed between participants with PD and control participants. These findings suggest that the immediate experiential impact of viewing artworks—at least in the context of a brief online viewing task—is broadly comparable between groups.

Nevertheless, descriptive patterns indicated somewhat greater variability in reported emotional and cognitive changes among participants with PD. Participants with PD appeared to report both increases and decreases in emotions such as sadness and anxiety more frequently than controls, and a larger proportion reported changes in cognitive symptoms. Since sample sizes are too small to compare means or to perform non-parametric testing for isolated symptom results per group, these descriptive trends should be interpreted cautiously. However, they may point toward greater fluctuation in affective or cognitive states during aesthetic experiences in individuals with PD.

Importantly, increases in reported emotional symptoms do not necessarily indicate negative experiences. Because emotional ratings included both positive and negative states, increases may reflect heightened emotional engagement rather than distress. In this sense, aesthetic experiences may still evoke meaningful emotional responses even in the absence of group-level differences in overall magnitude.

### 4.4. Limitations

Several limitations should be considered when interpreting the present findings. First, the study relied on online presentation of artworks using participants’ personal devices, which introduces variability in screen size, color reproduction, and viewing conditions. Future studies should replicate these findings under controlled laboratory conditions or in in-person exhibition settings (e.g., museums or galleries) to determine whether similar patterns hold when visual presentation and environmental factors are standardized in different contexts. Second, the cross-sectional design cannot address potential changes in aesthetic processing across disease progression as would be studied in a longitudinal design. In addition, considering that subject-ID was the strongest predictor, narrative-based approaches could capture individual context-dependent aspects of arts engagement more deeply. Third, although the sample size allowed for machine learning analyses of aesthetic judgments, some of the exploratory analyses of emotional interpretation and color associations may have been underpowered to detect subtle effects. Fourth, controls were partly recruited from partners and caregivers of individuals with PD, which may introduce shared environmental or socio-cultural influences and reduce group independence. In particular, because caregivers often share the daily environment and routines of individuals with PD, their aesthetic and emotional responses may partially converge with those of the PD participants, potentially attenuating between-group differences and thereby inflating the null findings’ directionality. Future studies should therefore include independent community-based control samples. Both samples also showed some imbalance in gender distribution between groups, which may limit generalizability, although gender did not significantly contribute to aesthetic predictions in the present analyses. Finally, the experiential symptom measures relied on self-report and therefore capture subjective perceptions rather than objective changes.

## 5. Conclusions

In summary, the present study provides evidence that aesthetic evaluation of visual artworks remains largely preserved in individuals with PD. While subtle differences in emotional interpretation and color-emotion associations may occur, overall patterns of liking and beauty are primarily shaped by individual taste and artwork characteristics rather than by disease status.

These findings suggest that, for screen-based and online art engagement, adaptation of visual content may not be necessary. However, given that the present study was conducted using digital stimuli, further research is needed to determine whether similar patterns extend to in-person museum environments, where additional spatial and sensory factors may influence aesthetic experience. Accordingly, efforts to improve cultural accessibility for individuals with PD may benefit from focusing on reducing practical and contextual barriers to engagement, while considering the specific characteristics of the viewing setting.

## Figures and Tables

**Table 1:** Demographics and clinical characteristics of the study population (*N* = 136).

Characteristic			PD (*n* = 87)	Aged-Matched Control (*n* = 49)
Age in years, mean ± SD			72 ± 6.7	73 ± 8.1
Gender identity (men), *n* (%)			50 (58)	18 (37)
Country of origin (the Netherlands), *n* (%)			87 (100)	49 (100)
Level of education, *n* (%) ^a^				
	Low		11 (13)	6 (12)
	Medium		26 (30)	12 (25)
	Higher		50 (58)	31 (63)
Primary diagnosis, *n* (%)				
	PD		81 (93)	NA
	Vascular parkinsonism		1 (1)	NA
	Unclear		5 (6)	NA
Hoehn and Yahr scale (HY score), *n* (%) ^b^				
	1		21 (24)	NA
	2		28 (32)	NA
	3		20 (23)	NA
	4		13 (15)	NA
	5		5 (6)	NA
MoCa-score, mean ± SD ^c^			19 ± 2.2	NA
Living situation ^d^				
	Alone		15 (17)	1 (2)
	With partner		65 (75)	48 (98)
	In facilitated care		3 (3)	NA
Working status ^e^				
	Not-working		78 (90)	34 (69)
	Working		9 (10)	15 (31)
		Paid	3 (33)	11 (73)
Perceived health status, *n* (%)				
	Excellent		NA	3 (6)
	Very good		NA	8 (16)
	Good		NA	30 (61)
	Fair		NA	8 (16)
Caregiving frequency, *n* (%)				
	24 h		NA	14 (29)
	Daily		NA	11 (22)
	3–6/week		NA	6 (12)
	1–2/week		NA	4 (8)
	<1/week		NA	1 (2)
	<1/month		NA	1 (2)
	Inconsistent		NA	9 (18)
	NA		NA	3 (6)

Note. ^a.^ Education was categorized according to the Dutch education system into three levels: low (primary and lower secondary education), medium (upper secondary and vocational education), and high (higher professional education and University), as well as an unknown category. ^b.^ Hoehn and Yahr (HY) score followed the standard scale and parameters for physical severity of the disease, see for details Table S1 in Supplementary Materials; ^c.^ Cognition was measured using the Montreal Cognitive Assessment (MoCA) short version (maximum 22 points). ^d.^ Living situation used the three items: ‘alone’ (“I live alone.”), ‘with partner or family’ (“I live with my partner/my partner and children/family member other than partner”), or ‘in facilitated care’ (“I live in an institution/independently and receive outpatient supervision from a residential or welfare organization (assisted living)/a house belonging to a residential or welfare organization (sheltered living). ^e.^ Working status was categorized as ‘working’ (full-time employment, part-time employment, self-employed, education following not paid by employer, voluntary work) or ‘not-working’ (retired, unemployed, disabled, sickness benefit, active in household).

**Table 2:** Predictive performance of machine learning models for aesthetic judgments.

Outcome	Prediction R^2^ (Mean ± SD)	Median R^2^	Shuffled R^2^	MAE (Mean ± SD)	Shuffled MAE	*p*-Value
Liking	0.317 ± 0.020	0.317	−0.035 ± 0.020	3.70 ± 0.10	4.64 ± 0.12	<0.001
Beauty	0.341 ± 0.020	0.331	−0.034 ± 0.023	3.48 ± 0.08	4.45 ± 0.13	<0.001

Note: Model performance was evaluated using nested cross-validation; shuffled models were generated by random permutation of target labels to estimate chance-level performance.

## Data Availability

The datasets generated and/or analyzed during the current study are available in the data repository: https://github.com/univiemops/aesthetic-preferences-in-pd (accessed on 8 June 2026).

## Supplementary Materials

The following supporting information can be downloaded at https://www.mdpi.com/article/10.3390/jcm15134865/s1, Table S1. Selection of artworks from the Vienna Art Picture System (VAPS). Table S2. Results selection of artworks along mean lightness and chroma values. Table S3. Group differences in dimensional emotion ratings for Self-Portrait 1 (Armand Henrion). Table S4. Group differences in dimensional emotion ratings for Self-Portrait 2 (Armand Henrion). Table S5. Group differences in dimensional emotion ratings for Self-Portrait 3 (Armand Henrion). Table S6. Group differences in dimensional emotion ratings for Self-Portrait 4 (Armand Henrion). Table S7. Group differences in dimensional emotion ratings for Self-Portrait 5 (Armand Henrion). Table S8. Group differences in categorical emotion selection (Geneva Emotion Wheel) and color choice across Henrion self-portraits. Table S9. Color selection. Table S10. Weighted Proportions of Color Associations by Emotion Category and Group. Table S11. Calculations of the weighted proportions of Color Associations by Emotion Category and Group (Table S10). Table S12. Between subject comparison of symptom changes per direction: increases. Table S13. Between subject comparison of symptom changes per direction: decreases. Table S14. Between subject comparison of absolute symptom changes. Figure S1. Self-portrait 1 of Armand Henrion. Figure S2. Self-portrait 2 of Armand Henrion. Figure S3. Self-portrait 3 of Armand Henrion. Figure S4. Self-portrait 4 of Armand Henrion. Figure S5. Self-portrait 5 of Armand Henrion. Figure S6. Color-emotion choices per category (positive-high control, positive-low control, negative-high control, negative-low control) for self-portrait 1 of Armand Henrion. Figure S7. Color-emotion choices per category (positive-high control, positive-low control, negative-high control, negative-low control) for self-portrait 2 of Armand Henrion. Figure S8. Color-emotion choices per category (positive-high control, positive-low control, negative-high control, negative-low control) for self-portrait 3 of Armand Henrion. Figure S9. Color-emotion choices per category positive-high control, positive-low control, negative-high control, negative-low control) for self-portrait 4 of Armand Henrion. Figure S10. Color-emotion choices per category (positive-high control, positive-low control, negative-high control, negative-low control) for self-portrait 5 of Armand Henrion. Figure S11. Color-emotion choices per category (positive-high control, positive-low control, negative-high control, negative-low control) for self-portraits 1–5 together of Armand Henrion. Note: Participants were allowed to select multiple emotions for each picture but were restricted to a single color choice per picture. To ensure each participant contributed equally to the group distribution regardless of the number of emotions selected, weighted proportions were calculated by assigning a total weight of 1.0 to each participant’s aggregate selections. Figure S12. N symptom change reported = 64 (Control *n* = 14; PD *n* = 50). Participants that reported experiencing “no change” are excluded from this calculation. Figure S13. N total symptom change reported = 93 (Control *n* = 24, PD *n* = 69). Participants that reported experiencing “no change” are excluded from this calculation. Figure S14. N total symptom change reported = 93 (Control *n* = 33, PD *n* = 75). Participants that reported experiencing “no change” are excluded from this calculation. Figure S15. *N* total symptom change reported (only PD *n* = 27). Participants that reported experiencing “no change” are excluded from this calculation. Figure S16. Percentage of participants experience changes by domain. Supplementary File S1. Data_control factor. Supplementary File S2. Data_valence factor. Supplementary File S3. full_Precision_Emotion_Colour_Analysis.
