# Supplementary material for: Preserved Aesthetic Judgements in Parkinson’s Disease: A Case–Control Study Suggests Limited Need for Content Adaptation for Receptive Arts Engagement
Source: J Clin Med. 2026 Jun 23;15(13):4865. doi: 10.3390/jcm15134865 (PMC13362079; doi:10.3390/jcm15134865)
Supplement: Supplementary file 1 [file jcm-15-04865-s001.zip › supplementary materials.pdf]

Supplementary Materials

Preserved Aesthetic Preferences in Parkinson's Disease: A Case-Control Study Suggests No Need for Content Adaptation for Receptive Arts Engagement

Blanca T.M. Spee[1,2*], Domicele Jonauskaite[3], Bastiaan R. Bloem[1], Emmy van den Berg[1], Nina Verhoeven[1], Dagne Bagdonaviciute[1], Nicolien Dam[1], Julia S. Crone[2,4], Jorik Nonnekes[5], David Steyrl[2], Matthew Pelowski[2]

[1] Department of Neurology, Radboud University Medical Center; Donders Institute for Brain, Cognition and Behavior; Center of Expertise for Parkinson & Movement Disorders; Nijmegen, The Netherlands

[2] Department of Cognition, Emotion, and Methods in Psychology, Faculty of Psychology, University of Vienna, Vienna, Austria

[3] Institute of Psychology, University of Lausanne, Lausanne, Switzerland

[4] Centre for Cognitive Neuroscience, Department of Psychology, Paris Lodron University Salzburg, Salzburg, Austria

[5] Department of Rehabilitation, Radboud University Medical Center; Donders Institute for Brain, Cognition and Behavior; Center of Expertise for Parkinson & Movement Disorders; Nijmegen, The Netherlands

* Corresponding Author: blanca.spee@univie.ac.at; blanca.spee@radboudumc.nl

Supplementary Tables

Table S1. Selection of artworks from the Vienna Art Picture System (VAPS)

Picture number in VAPS	Name of artist	Date	Title	Epoch/Style	Category
'10204.jpg'	Caravaggio	±1610	The Denial of Saint Peter	Baroque and Rococo	Scenes
'10207.jpg'	Pier Francesco Mazzechelli	1610	Jacob Wrestling the Angel	Baroque and Rococo	Scenes
'10249.jpg'	Joseph Wright of Derby	1765	At the Light of a Candle, Three Men Study a Small Replica of the Borghese Gladiator	Baroque and Rococo	Scenes
'10514.jpg'	Max Slevogt	1904	The Dancer Marietta di Rigardo	Impressionistic tendencies	Scenes
'10603.jpg'	Maximilien Luce	1890	Morning, Interior	Post-impressionistic tendencies	Scenes
'10612.jpg'	Paul Cézanne	1895-1906	The Large Bathers (Les Grandes Baigneuses)	Post-impressionistic tendances	Scenes
'10708.jpg'	Max Backmann	1909	Scene from the Desctruction of Messina	Expressionistic tendencies	Scenes
'10713.jpg'	Max Pechstein	1913	Fishing Boat	Expressionistic tendencies	Scenes
'10721.jpg'	Gabriele Münter	1908	Jawlensky and Werefkin	Expressionistic tendencies	Scenes
'10802.jpg'	André hote	1917	Rugby (Les Joueurs de Rugby)	Cubistic tendencies	Scenes
'10805.jpg'	Pablo Picasso	1907	The Brothel of Avignon (Les Demoiselles d'Avignon)	Cubisitic tendencies	Scenes
'10812.jpg'	George Grosz	1916-1917	The City	Cubistic tendencies	Scenes
'30212.jpg'	Jan van Goyen	1642	A Windmill by a River	Baroque and Rococo	Landscape
'30220.jpg'	Bernardo Bellotto	1748	View of Dresden from the left Bank of the Elbe, below the Fortifications	Baroque and Rococo	Landscape
'30224.jpg'	Bernardo Bellotto	1735	The Scuola di San Rocco	Baroque and Rococo	Landscape
'30508.jpg'	Pierre-Auguste Renoir	1875	The Grands Boulevards	Impressionistic tendencies	Landscape

'30513.jpg'	Max Slevogt	1927	Herbstlandschaft bei Neukastel	Impressionistic tendencies	Landscape
'30618.jpg'	Vincent van Gogh	1885	Landscape with Canal	Post-impressionistic tendencies	Landscape
'30709.jpg'	Maurce de Vlaminck	1905	Restaurant de la Machine at Bougival	Expressionistic tendencies	Landscape
'30711.jpg'	Erich Heckel	1918	Springtime	Expressionistic tendencies	Landscape
'30716.jpg'	Max Pechstein	1922	Melting Snow	Expressionistic tendencies	Landscape
'30801.jpg'	Georges Braque	1908	Road near L'Estaque	Cubistic tendencies	Landscape
'30805.jpg'	Diego Rivera	1913	Spanisch Landscape	Cubistic tendencies	Landscape
'30807.jpg'	Diego Rivera	1913	Trees and Walls in Toledo	Cubistic tendencies	Landscape
'50804.jpg'	Albert Gleizes	1916	On a Sailboat	Cubistic tendencies	Toward abstraction
'50805.jpg'	Fernard Léger	1921	Mechanical Element	Cubistic tendencies	Toward abstraction
'50815.jpg'	František Kupka	1912	Untitled	Cubistic tendencies	Toward abstraction
'50911.jpg'	Kaximir Malevich	1915	Supremus No. 50	Constructivist tendencies	Toward abstraction
'50925.jpg'	Josef Albers	1931	Steps	Constructivist tendencies	Toward abstraction
'50939.jpg'	Richard Anuszkiewicz	1963	Squaring the Circle	Constructivist tendencies	Toward abstraction
'51103.jpg'	Arshile Gorky	1944	The Liver is the Cock's Comb	Surrealistic tendencies	Toward abstraction
'51109.jpg'	Yves Tanguy	1942	Slowly Toward the North	Surrealistic tendencies	Toward abstraction
'51113.jpg'	Joan Miró	1926	Person Throwing a Stone at a Bird	Surrealistic tendencies	Toward abstraction
'51315.jpg'	Mark Tobey	1964	Advance of History	Abstract Expressionistic tendencies	Toward abstraction
'51327.jpg'	Mark Rothko	1960	Untitled	Abstract Expressionistic tendencies	Toward abstraction
'51335.jpg'	Jasper Johns	1961	Map	Abstract Expressionistic tendencies	Toward abstraction

Table S2. Results selection of artworks along mean lightness and chroma values

Picture ID in VAPS	Mean Lightness	Mean Chroma
10204	17.597	10.131
10207	23.519	13.922
10249	10.751	7.389
10514	35.629	17.297
10603	44.268	18.355
10612	31.848	11.794
10708	31.715	11.605
10713	47.464	18.490
10721	41.350	33.251
10802	58.256	15.824
10805	59.375	18.325
10812	34.929	31.223
30212	55.178	9.896
30220	61.856	15.685
30224	48.456	16.882
30508	42.677	9.280
30513	45.847	19.393
30618	39.162	28.410
30709	46.511	37.993
30711	50.849	26.530
30716	45.443	20.985
30801	47.941	26.695
30805	55.171	28.330
30807	63.313	23.579
50804	39.647	24.175
50805	49.357	13.212
50815	62.517	35.922
50911	58.611	14.166
50925	21.307	1.168
50939	75.768	58.020
51103	55.066	22.789
51109	16.058	15.057
51113	46.340	36.628
51315	54.079	14.460
51327	52.934	31.895
51335	61.054	30.059

Table S3. Group differences in dimensional emotion ratings for Self-Portrait 1 (Armand Henrion).

	M (control *n*=47) ± SD	*M* (PD *n*=83) ± SD	*t*	*df*	*p*	CI (95%)	Cohen's *d*
P1 (positive_valence)	7.234±1.549	6.711±1.897	1.703	111.97	.091	[-0.086,1.132]	0.29
P1 (negative_valence)	1.894±2.129	2.169 ±2.053	-0.717	92.777	.475	[-1.037,0.487]	-0.13
P1 (control)	5.085±2.474	4.373 ±2.626	1.541	100.46	.127	[-0.205, 1.628]	0.28
P1 (lack of control)	2.915±2.448	3.060±2.416	-0.327	94.6	.745	[-1.028, 0.738]	-0.06
P1 (arousal_active)	3.617±2.634	3.542±2.643	0.155	95.906	.877	[-0.881, 1.030]	0.03
P1 (arousal_ calm)	5.170±2.316	5.145±2.430	0.060	99.552	.953	[-0.828, 0.880]	0.01

Table S4. Group differences in dimensional emotion ratings for Self-Portrait 2 (Armand Henrion).

	M (control *n*=47) ± SD	*M* (PD *n*=83) ± SD	*t*	*df*	*p*	CI (95%)	Cohen's *d*
P2 (positive_valence)	5.936±2.068	5.272±2.106	1.734	97.088	.075	[-0.071, 1.438]	0.33
P2 (negative_valence)	3.085±2.292	3.542±2.591	-1.041	105.64	.300	[-1.327, 0.413]	-0.18
P2 (control)	4.936±2.344	3.952±2.527	2.236	101.75	**.028***	[0.111, 1.858]	0.40
P2 (lack of control)	3.447±2.273	3.614 ±2.517	-0.389	103.97	.699	[-1.024, 0.688]	-0.07
P2 (arousal_active)	4.128±2.626	4.120 ±2.698	0.015	97.811	.988	[-0.954, 0.968]	0.003
P2 (arousal_ calm)	4.277±2.310	3.880±2.205	0.957	91.997	.341	[-0.427, 1.221]	0.18

* significant, alpha < .05.

Table S5. Group differences in dimensional emotion ratings for Self-Portrait 3 (Armand Henrion).

	M (control *n*=47) ± SD	M (PD *n*=83) ± SD	*t*	*df*	*p*	CI (95%)	Cohen's *d*
P3 (positive_valence)	1.340±1.710	1.807 ±1.565	-1.541	88.783	.127	[-1.069, 0.135]	-0.29
P3 (negative_valence)	7.170±2.099	6.639±2.540	1.284	111.09	.202	[-0.289, 1.352]	0.22
P3 (control)	3.383±2.112	2.627±1.879	2.041	86.708	**.044***	[0.020, 1.493]	0.38
P3 (lack of control)	4.872±2.667	4.928±2.815	-0.111	99.997	.912	[-1.041, 0.930]	-0.02
P3 (arousal_active)	3.596±2.716	3.711±2.712	-0.232	95.522	.817	[-1.099, 0.869]	-0.04
P3 (arousal_ calm)	1.872±1.849	1.904±1.559	-0.098	82.944	.922	[-0.667, 0.604]	-0.02

* significant, alpha < .05.

Table S6. Group differences in dimensional emotion ratings for Self-Portrait 4 (Armand Henrion).

	M (control *n*=47) ± SD	M(PD *n*=83) ± SD	*t*	*df*	*p*	CI (95%)	Cohen's *d*
P4 (positive_valence)	6.532±1.381	6.289±2.021	0.810	123.43	.419	[-0.350, 0.836]	0.13
P4 (negative_valence)	2.106±1.684	2.337±2.088	-0.700	109.9	.486	[-0.885, 0.423]	-0.12
P4 (control)	4.894±2.258	4.675±2.705	0.494	110.31	.623	[-0.660, 1.098]	0.09
P4 (lack of control)	3.787±2.196	3.313±2.414	1.140	103.34	.257	[-0.351, 1.299]	0.20
P4 (arousal_active)	4.234±2.606	4.301±2.408	-0.145	89.494	.885	[-0.987, 0.853]	-0.03
P4 (arousal_ calm)	4.872±2.007	4.518 ±2.097	0.951	99.216	.344	[-0.385, 1.093]	0.17

Table S7. Group differences in dimensional emotion ratings for Self-Portrait 5 (Armand Henrion).

	M (control *n*=47) ± SD	*M* (PD *n*=83) ± SD	*t*	*df*	*p*	CI (95%)	Cohen's *d*
P5 (positive_valence)	1.830±2.057	1.711±1.330	0.356	68.214	.723	[-0.547, 0.785]	0.07
P5 (negative_valence)	7.702±1.876	7.024±2.230	1.847	109.71	.067	[-0.050, 1.406]	0.32
P5 (control)	3.660±2.306	3.566±2.312	0.221	95.866	.825	[-0.743, 0.930]	0.04
P5 (lack of control)	4.915±2.448	4.229±2.619	1.496	101.14	.138	[-0.223, 1.595]	0.27
P5 (arousal_active)	3.298±2.493	3.458±2.596	-0.346	98.921	.730	[-1.077, 0.757]	-0.06
P5 (arousal_ calm)	1.915±2.009	2.277±1.720	-1.039	83.999	.302	[-1.055, 0.331]	-0.20

Table S8. Group differences in categorical emotion selection (Geneva Emotion Wheel) and color choice across Henrion self-portraits.

	Colour		Emotion		Colour+ emotion	
	p	Cramer's *V*	*p*	Cramer's *V*	*p*	Cramer's *V*
Picture 1	.348	.295	.356	.095	.404	.392
Picture 2	.382	.293	**.045**	.158	.152	.478
Picture 3	.178	.325	.865	.044	.082	.423
Picture 4	.080	.352	.214	.113	.442	.431
Picture 5	.064	.343	.151	.135	.092	.453
Total	**.026**	.179	.380	.042	.617	.215

Note. p-values were derived from Fisher's Exact Tests using Monte Carlo simulation (5,000 iterations) to account for small, expected cell frequencies (cells with $n<5$). Effect sizes are reported as Cramer's V.

Table S9. Color selection

Color	Control (*N*)	Parkinson's (*N*)	Residual (*z*)	*p*
Red	34	49	0.98	.329
Orange	14	21	0.49	.626
Yellow	22	31	0.85	.397
Green	17	30	0.00	.998
Turquoise	6	22	-1.66	.097
Blue	36	45	1.66	.097
Purple	17	45	-1.51	.132
**Pink**	**23**	**8**	**2.95**	**.003**
Brown	27	45	-0.42	.673
**Gray**	**24**	**71**	**-1.92**	**.054**
Black	48	48	-0.53	.597

Note. N represents the total frequency of color selections across all five pictures. Residuals and p-values are calculated based on the distribution of these total selections reported from the Controls group's perspective. Significant associations ($p < .05$) are indicated in bold.

Table S10. Weighted Proportions of Color Associations by Emotion Category and Group

		Control (*n*=47)		Parkinson's (*n*=83)	
Emotion Category	Color Choice	*n*	Weighted Proportions	*n*	Weighted Proportions
High Control Positive	Red	19	5.90	28	4.60
	Yellow	13	4.38	19	3.12
	Blue	11	3.85	19	3.15
	Green	13	3.79	11	1.87
	Pink	9	2.92	6	0.91
	Gray	4	1.27	13	3.36
High Control Negative	Gray	14	4.20	26	5.55
	Black	12	3.76	22	4.23
	Brown	13	3.58	23	4.13
	Purple	7	2.18	18	3.70
	Red	7	2.74	13	2.01
Low Control Positive	Red	17	5.17	23	4.29
	Blue	13	4.86	23	4.25
	Yellow	10	2.91	16	2.39
	Orange	9	2.66	13	2.21
	Pink	8	2.61	5	0.76
Low Control Negative	Gray	16	4.89	27	5.69
	Blue	11	4.31	8	1.51
	Brown	14	4.26	21	3.80
	Black	11	3.40	20	3.72
	Purple	7	2.14	13	1.91

Note. Participants were allowed to select multiple emotions for each picture but were restricted to a single color choice per picture. To ensure each participant contributed equally to the group distribution regardless of the number of emotions selected, weighted proportions were calculated by assigning a total weight of 1.0 to each participant's aggregate selections.

Table S11. Calculations of the weighted proportions of Color Associations by Emotion Category and Group (table S10)

=	Positive		Negative		High control		Low control	
	p	Cramer's *V*	*p*	Cramer's *V*	*p*	Cramer's *V*	*p*	Cramer's *V*
All picutes	.207	0.213	.685	0.168	.708	0.133	.816	0.127

Note. *p*-values were derived from Fisher's Exact Tests

Table S12. Between subject comparison of symptom changes per direction: increases.

Increase	PD			control			95%	CI			
	Mean	*SD*	Range	Mean	*SD*	Range	*t*	Lower	Upper	*p*	Cohen's *d*
Cognitive	4.77	2.28	4.09-5.45	4.67	1.78	3.66, 5.67	-0.16	-1.42	1.73	.870	0.16
Emotional	4.24	2.08	3.74-4.75	4.68	2.19	3.77-5.6	-0.82	-0.69	0.28	.415	0.21
Motivational	5.12	2.1	4.62, 5.61	5.2	2.25	4.39, 6.01	-0.17	-0.47	0.39	.862	-0.04
Physical	3.31	2.32	2.04-4.57	-	-	-	-	-	-	-	-

Table S13. Between subject comparison of symptom changes per direction: decreases

Decrease	PD			control			95%	CI			
	Mean	*SD*	Range	Mean	*SD*	Range	*t*	Lower	Upper	*p*	Cohen's*d*
Cognitive	3.43	2.7	1.43-5.43	3	2.83	0.92 – 6.92	0.19	-1.4	1.73	.870	0.16
Emotional	5	3.46	1.08-8.92	2.5	0.70	1.52 - 3.48	-1.21	-2.74	0.99	.337	0.87
Motivational	3.33	2.5	1.33-5.34	5	2.65	2.01 - 7.99	-0.90	-0.76	2.07	.417	0.65
Physical	4	1.51	2.95-5.05	-	-	-	-	-	-	-	-

Table S14. Between subject comparison of absolute symptom changes.

Absolute change	PD			control			95%	CI			
	Mean	*SD*	Range	Mean	*SD*	Range	*t*	Lower	Upper	*p*	Cohen's *d*
Cognitive	4.58	2.36	(3.93, 5.23)	4.43	1.91	(3.43, 5.43)	0.25	-0.53	0.66	.805	-0.66
Emotional	4.28	2.13	(3.77, 4.78)	4.50	2.19	(3.63, 5.37)	-0.44	-0.57	0.36	.665	-0.11
Motivation	4.97	2.17	(4.48, 5.46)	5.18	2.24	(4.42, 5.95)	-0.45	-0.05	0.31	.655	-0.10
Physical	3.57	2.04	(2.7, 4.44)	-	-	-					

Note. Comparison of magnitude of change. No indication of value or direction of change. Change is reported on a scale from 1-10. Comparison tests: *t*-test and Wilcoxon test if *t*-test failed; if – then control group reported higher change. If + then Parkinson higher change.
Note that an increase in e.g. emotional symptoms can be both an increase in sadness as an increase in happiness, these results don't clarify the contextual value of the increased symptoms, only the mean magnitude of increased and decreased symptoms per domain. For insight in the sub-symptoms see the bar-plots below (Fig. S12-S16 in Supplementary Materials).

Supplementary Figures

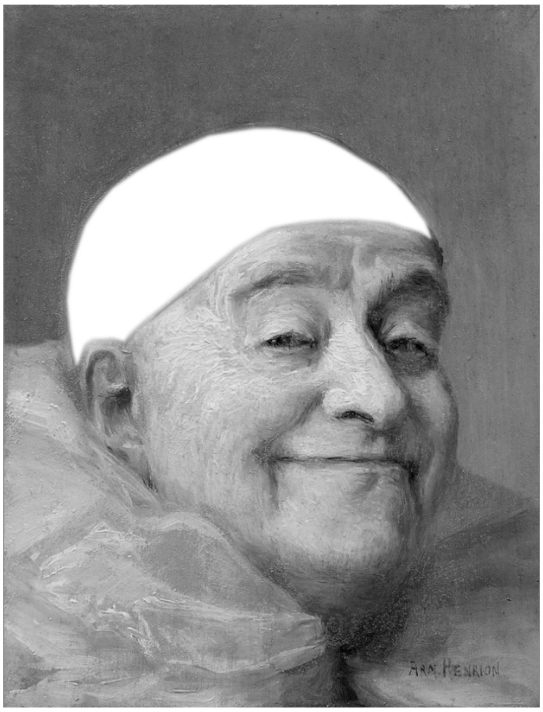

Figure S1. Self-portrait 1 of Armand Henrion

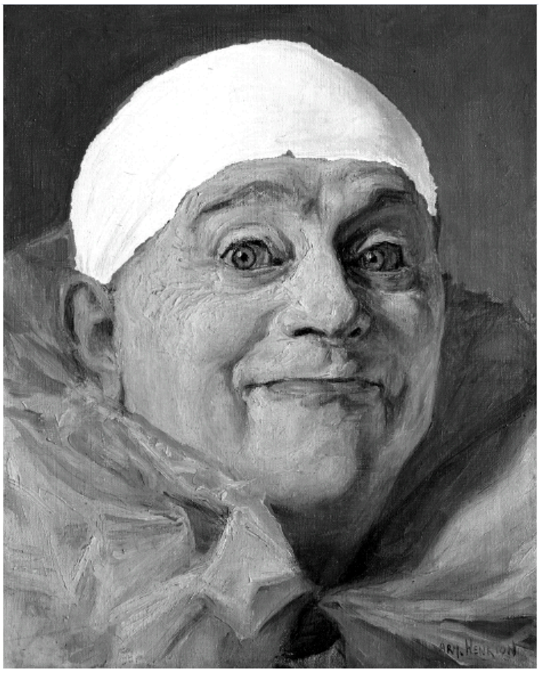

Figure S2. Self-portrait 2 of Armand Henrion

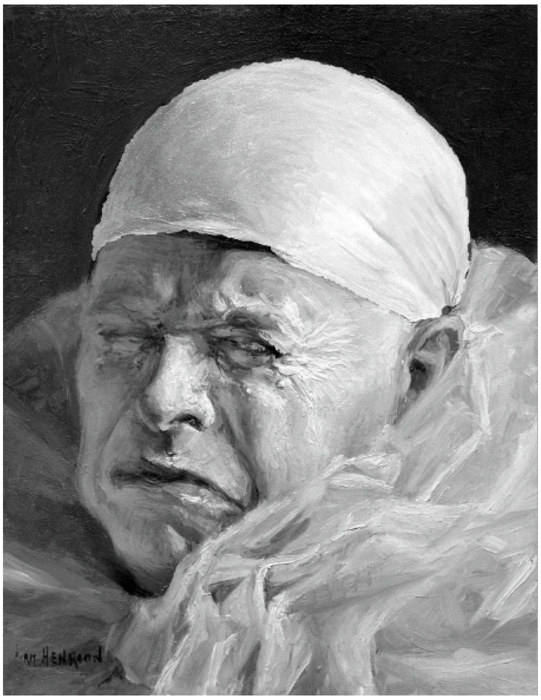

Figure S3. Self-portrait 3 of Armand Henrion

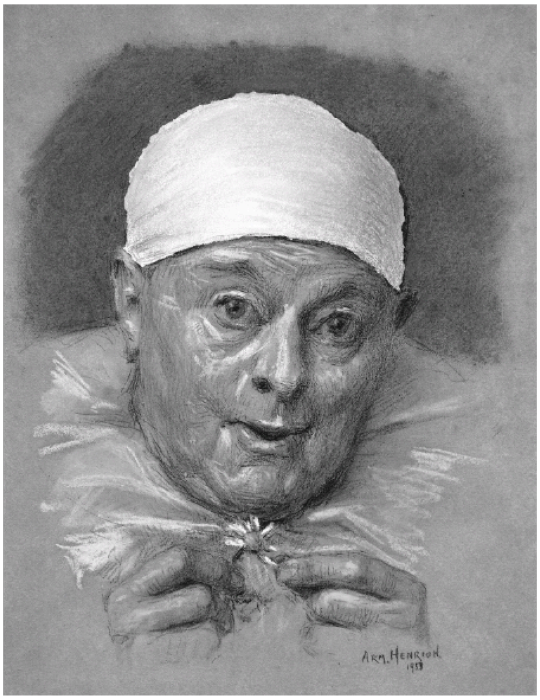

Figure S4. Self-portrait 4 of Armand Henrion

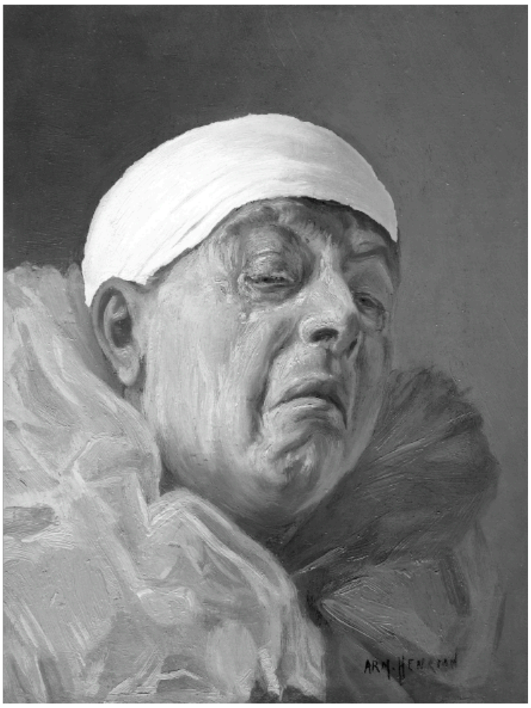

Figure S5. Self-portrait 5 of Armand Henrion

Picture 1

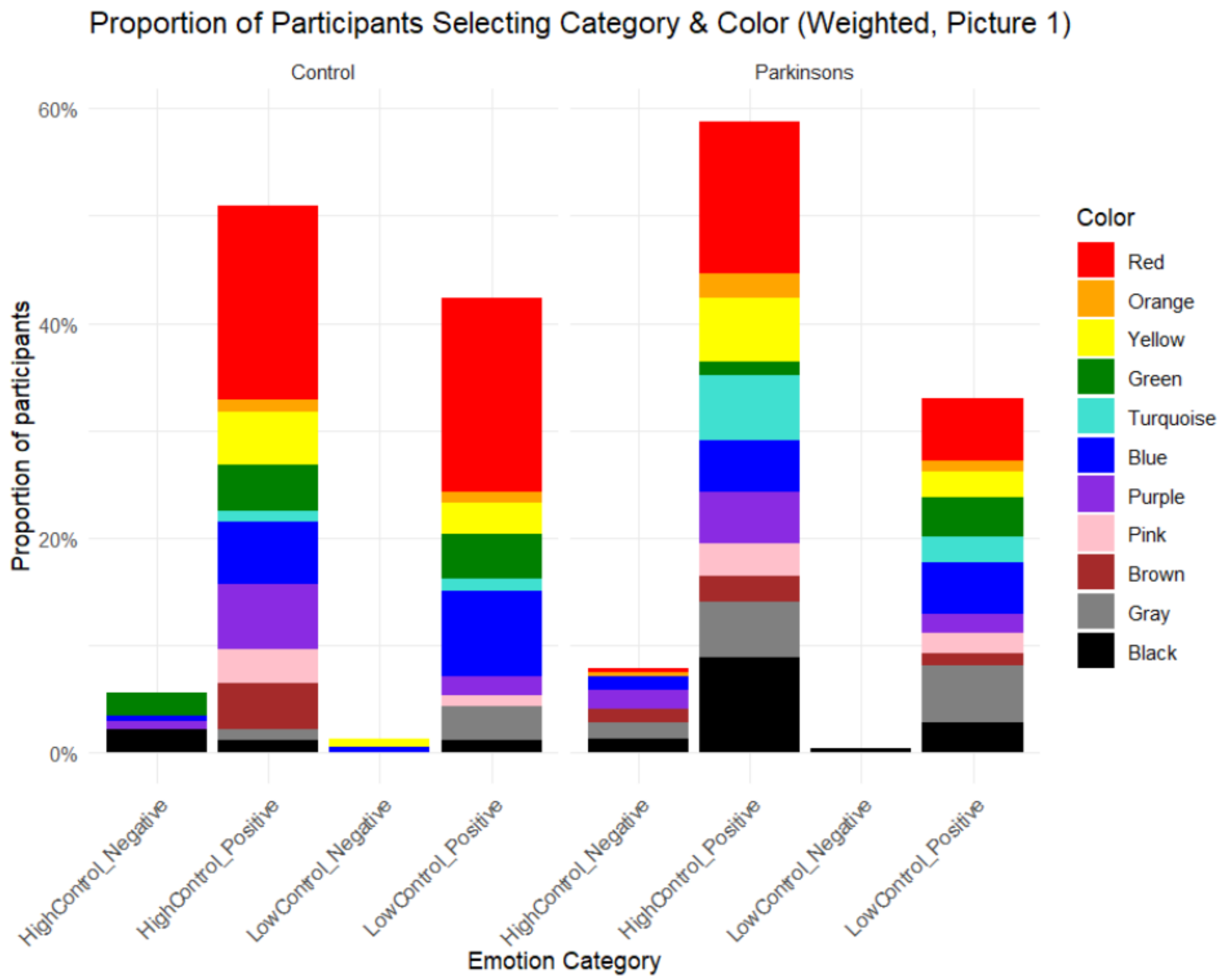

Figure S6. Color-emotion choices per category (positive-high control, positive-low control, negative-high control, negative-low control) for self-portrait 1 of Armand Henrion.

Picture 2

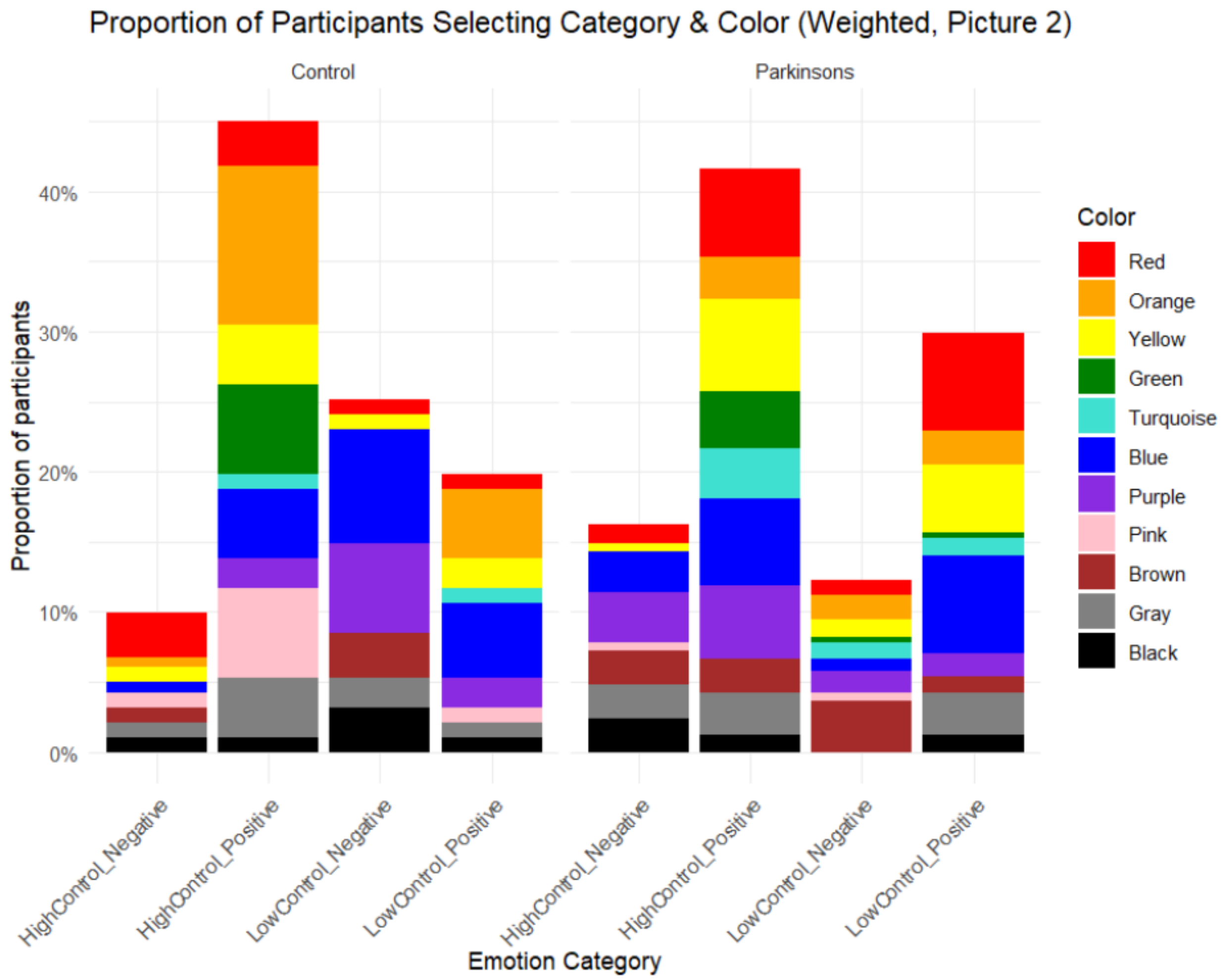

Figure S7. Color-emotion choices per category (positive-high control, positive-low control, negative-high control, negative-low control) for self-portrait 2 of Armand Henrion.

Picture 3

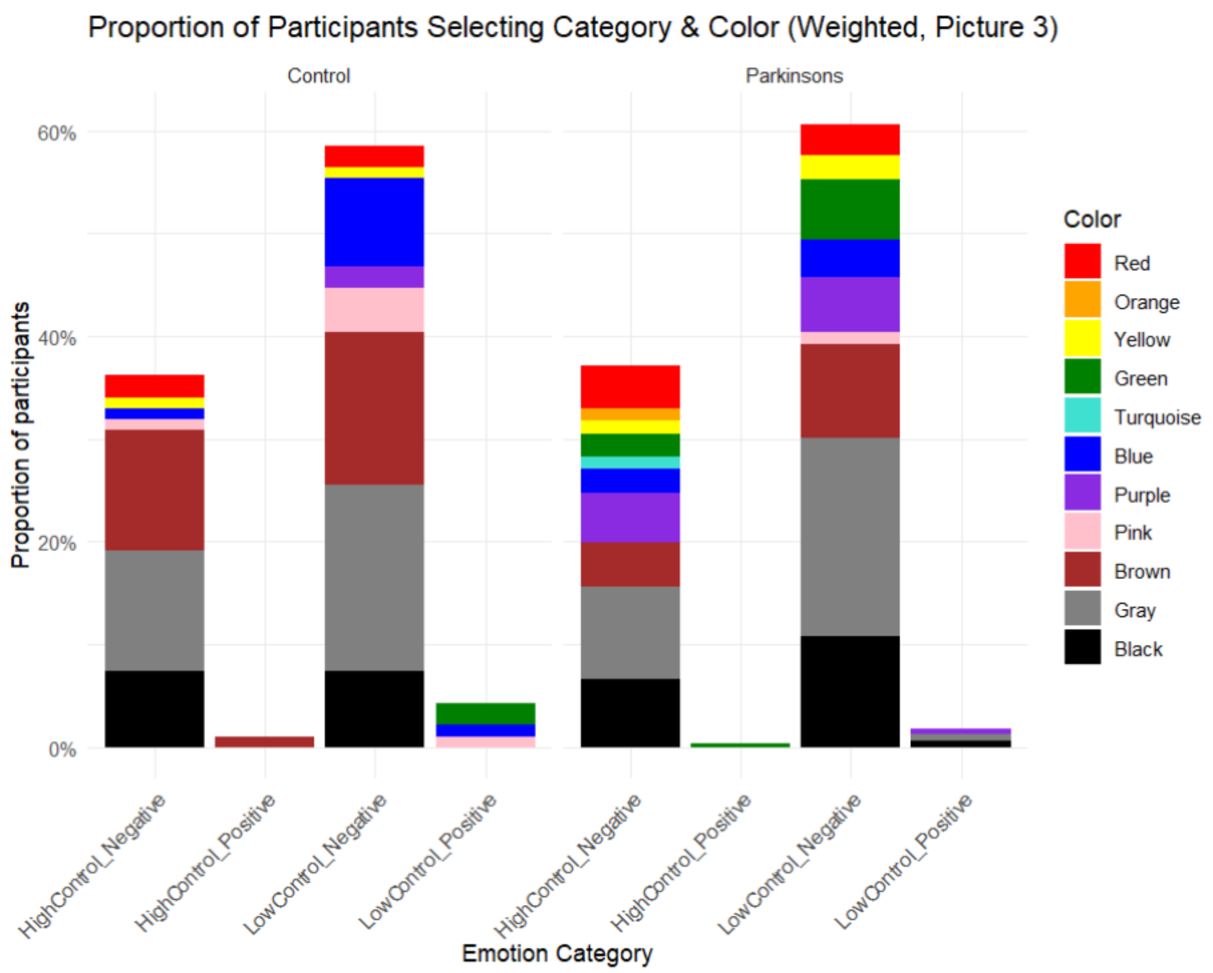

Figure S8. Color-emotion choices per category (positive-high control, positive-low control, negative-high control, negative-low control) for self-portrait 3 of Armand Henrion.

Picture 4

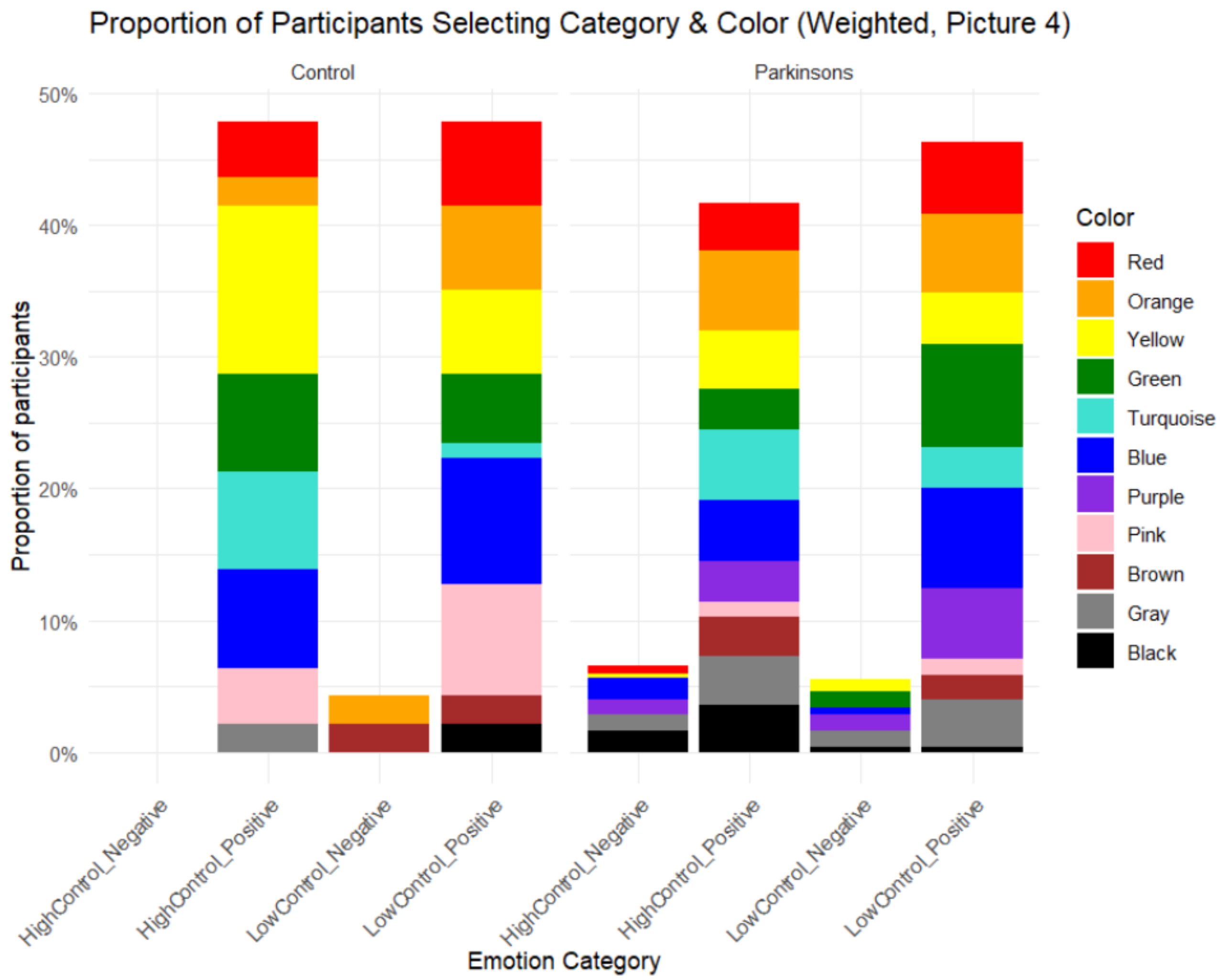

Figure S9. Color-emotion choices per category positive-high control, positive-low control, negative-high control, negative-low control) for self-portrait 4 of Armand Henrion.

Picture 5

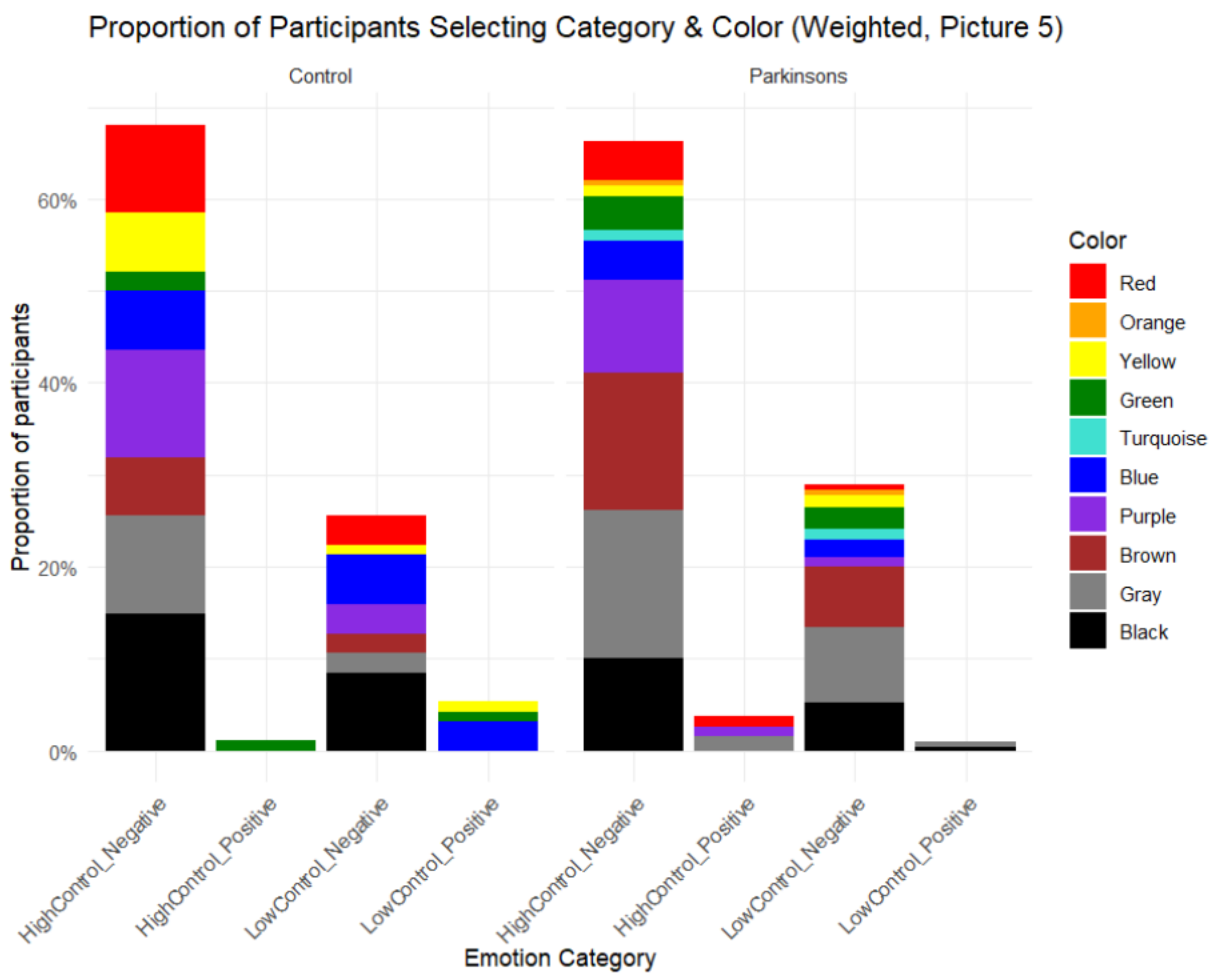

Figure S10. Color-emotion choices per category (positive-high control, positive-low control, negative-high control, negative-low control) for self-portrait 5 of Armand Henrion.

All Pictures

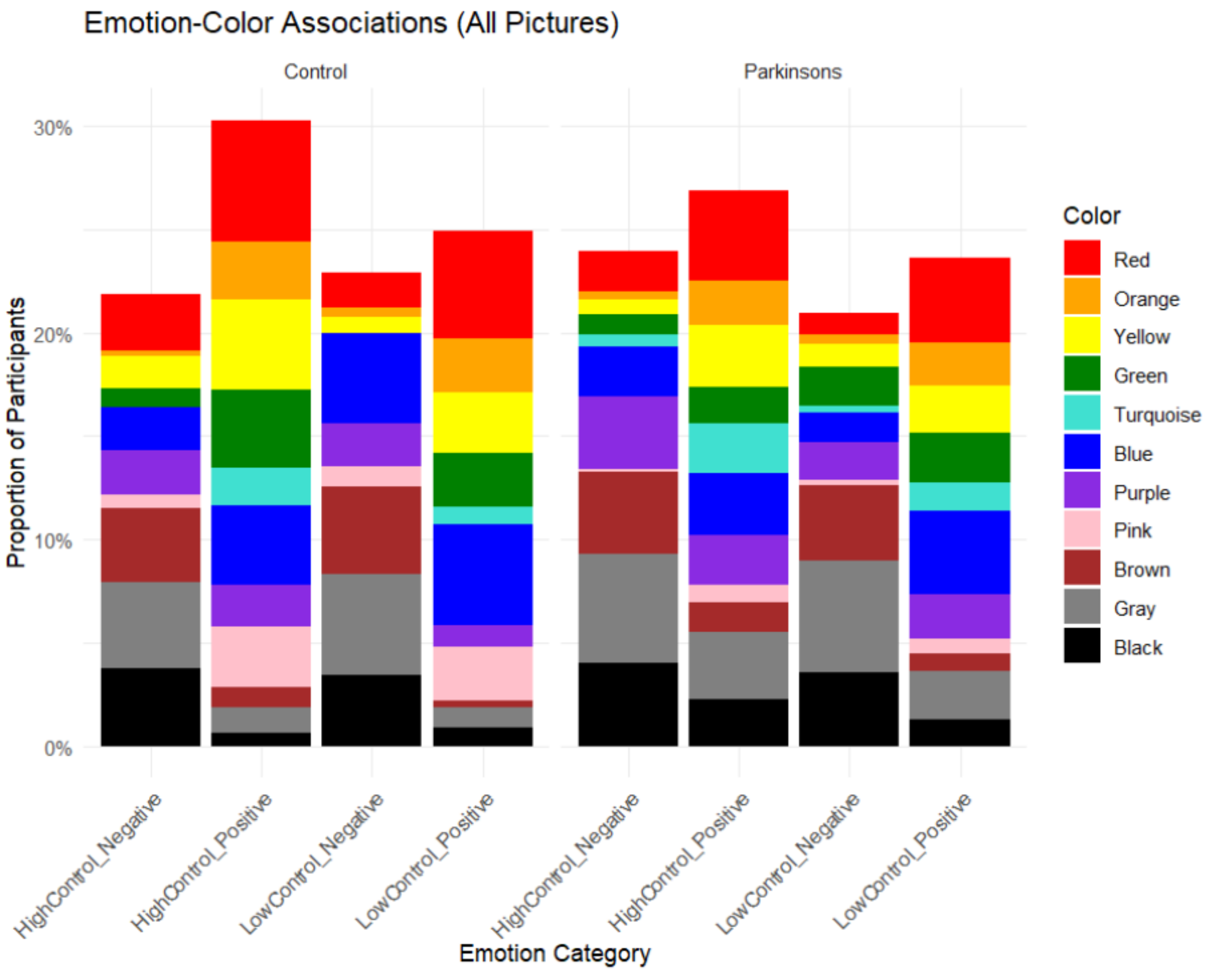

Figure S11. Color-emotion choices per category (positive-high control, positive-low control, negative-high control, negative-low control) for self-portraits 1-5 together of Armand Henrion. Note: Participants were allowed to select multiple emotions for each picture but were restricted to a single color choice per picture. To ensure each participant contributed equally to the group distribution regardless of the number of emotions selected, weighted proportions were calculated by assigning a total weight of 1.0 to each participant's aggregate selections.

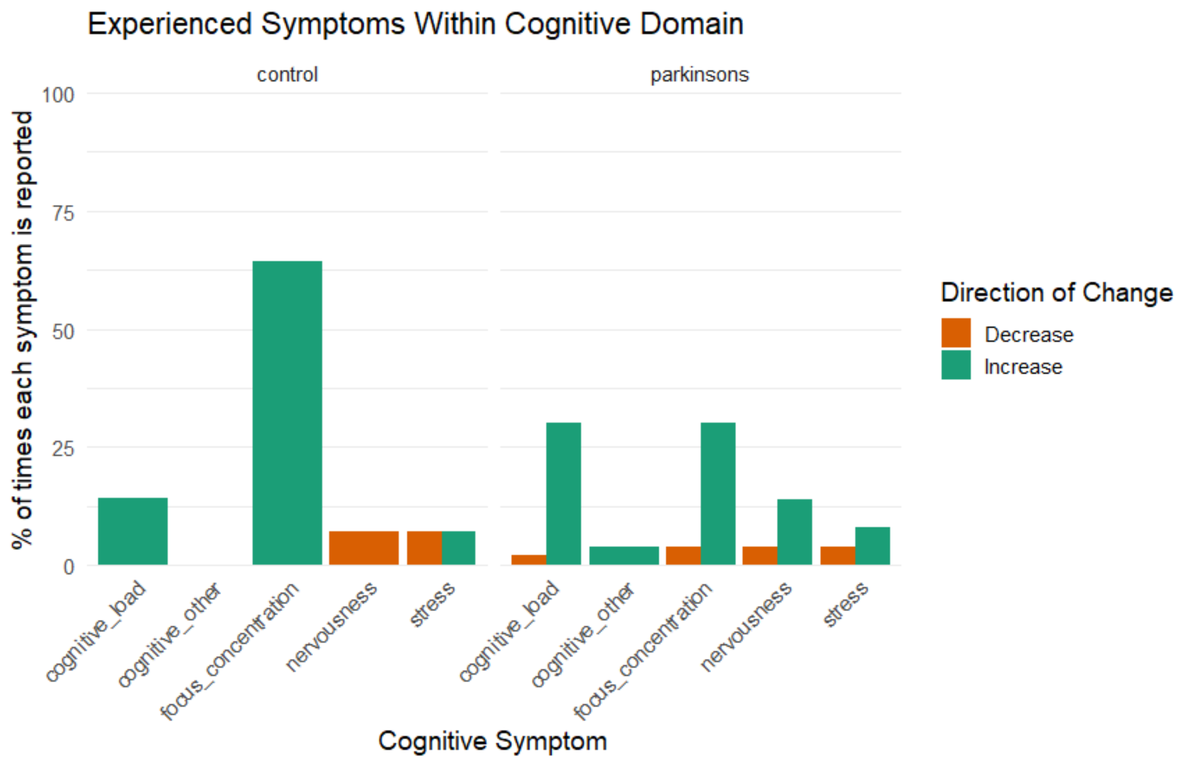

Figure S12. *N* symptom change reported = 64 (Control *n* = 14; PD *n* = 50). Participants that reported experiencing "no change" are excluded from this calculation.

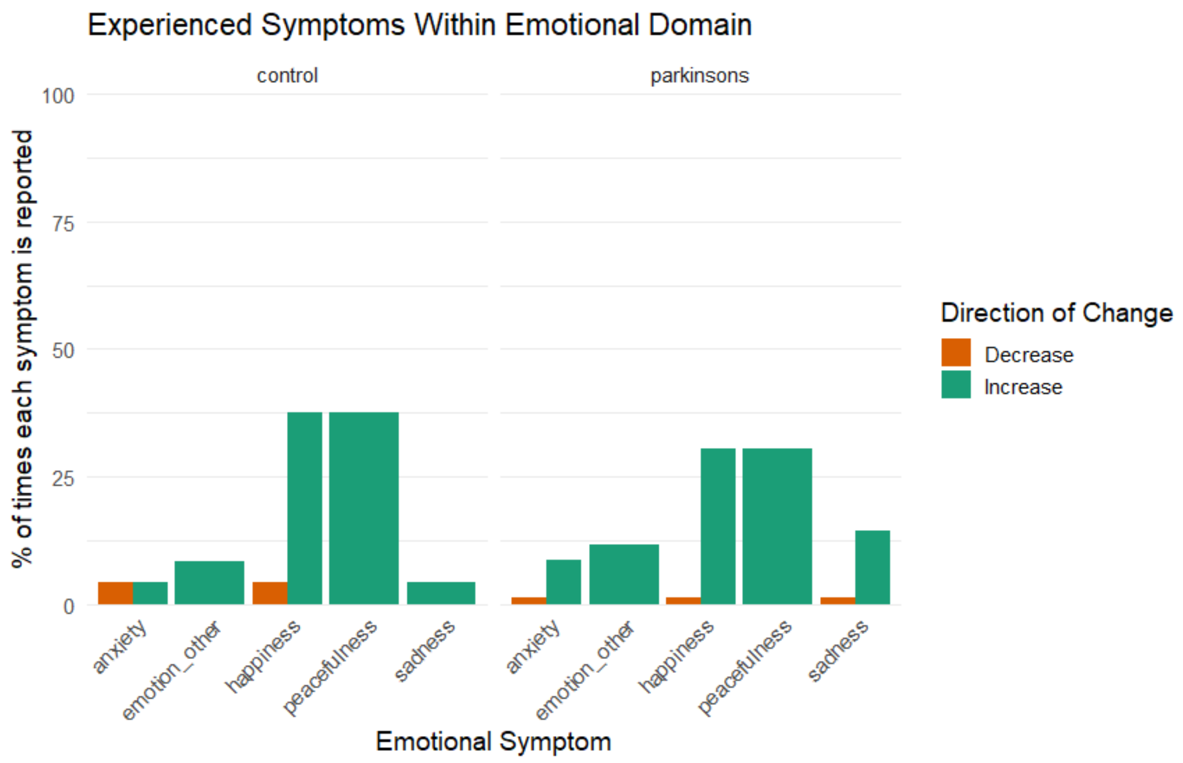

Figure S13. *N* total symptom change reported = 93 (Control *n* = 24, PD *n* = 69). Participants that reported experiencing "no change" are excluded from this calculation.

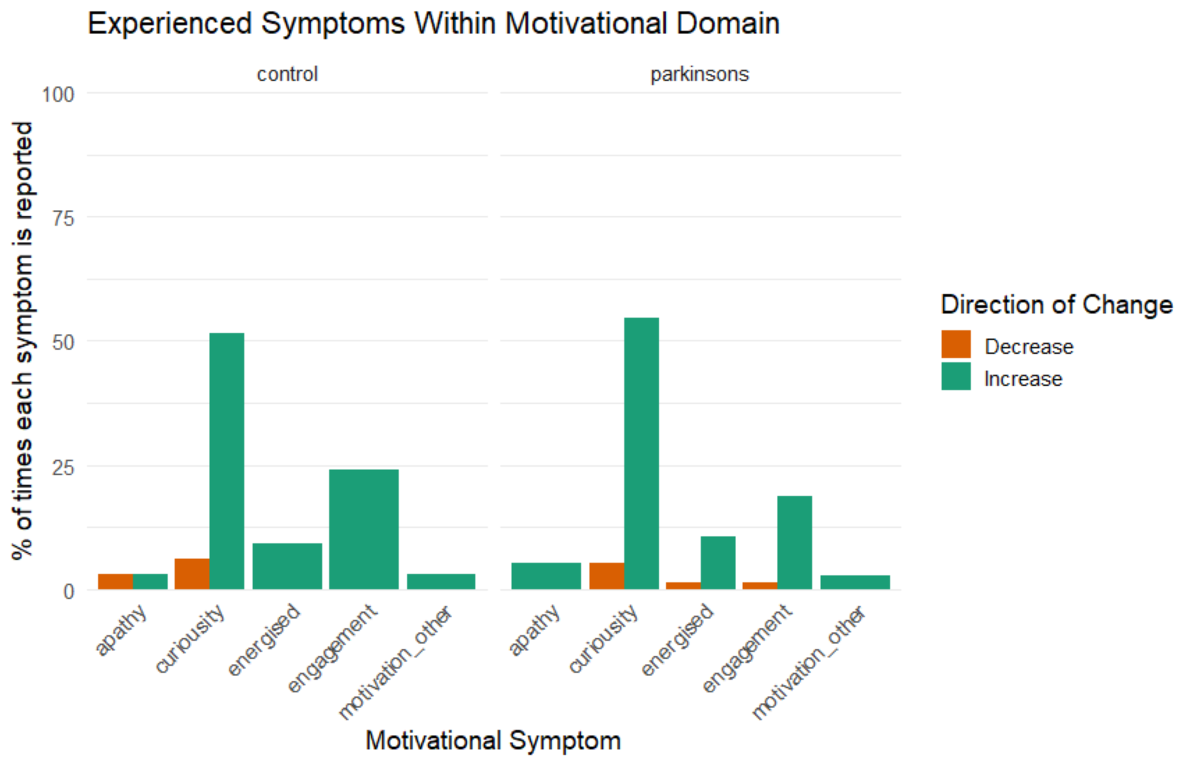

Figure S14. *N* total symptom change reported = 93 (Control *n* = 33, PD *n* = 75). Participants that reported experiencing “no change” are excluded from this calculation.

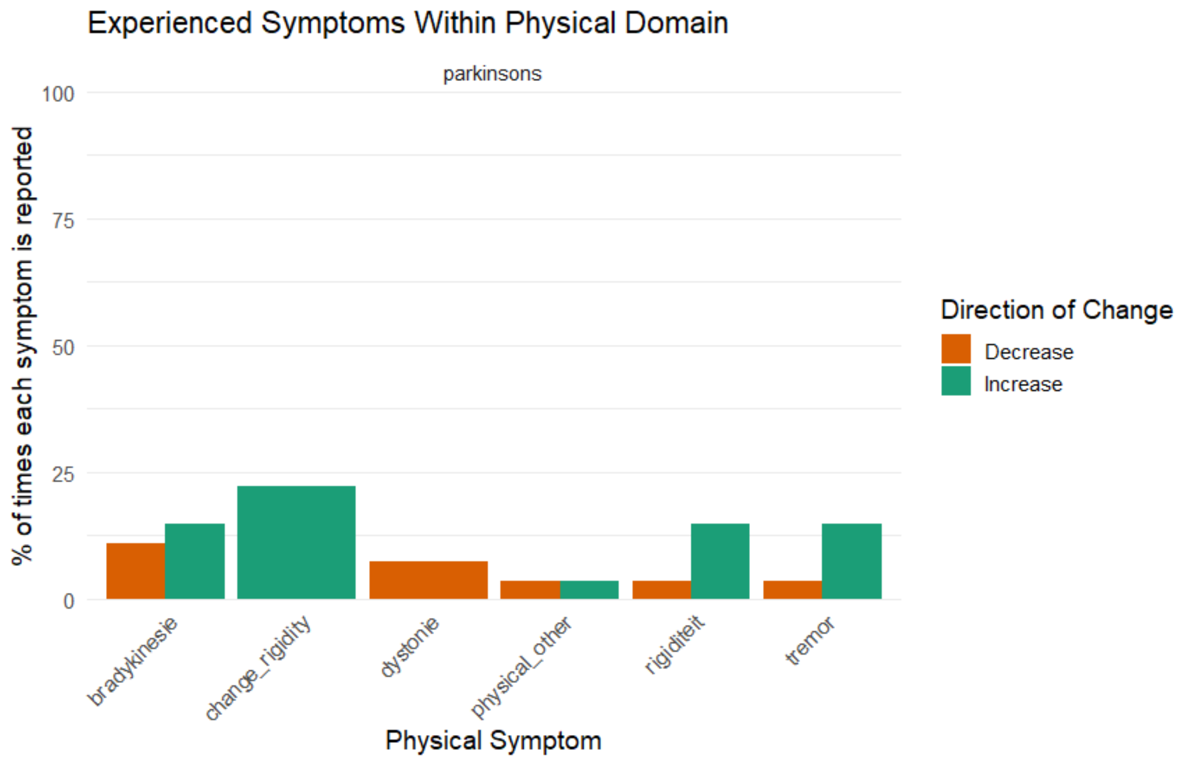

Figure S15. N total symptom change reported (only PD *n* = 27). Participants that reported experiencing “no change” are excluded from this calculation.

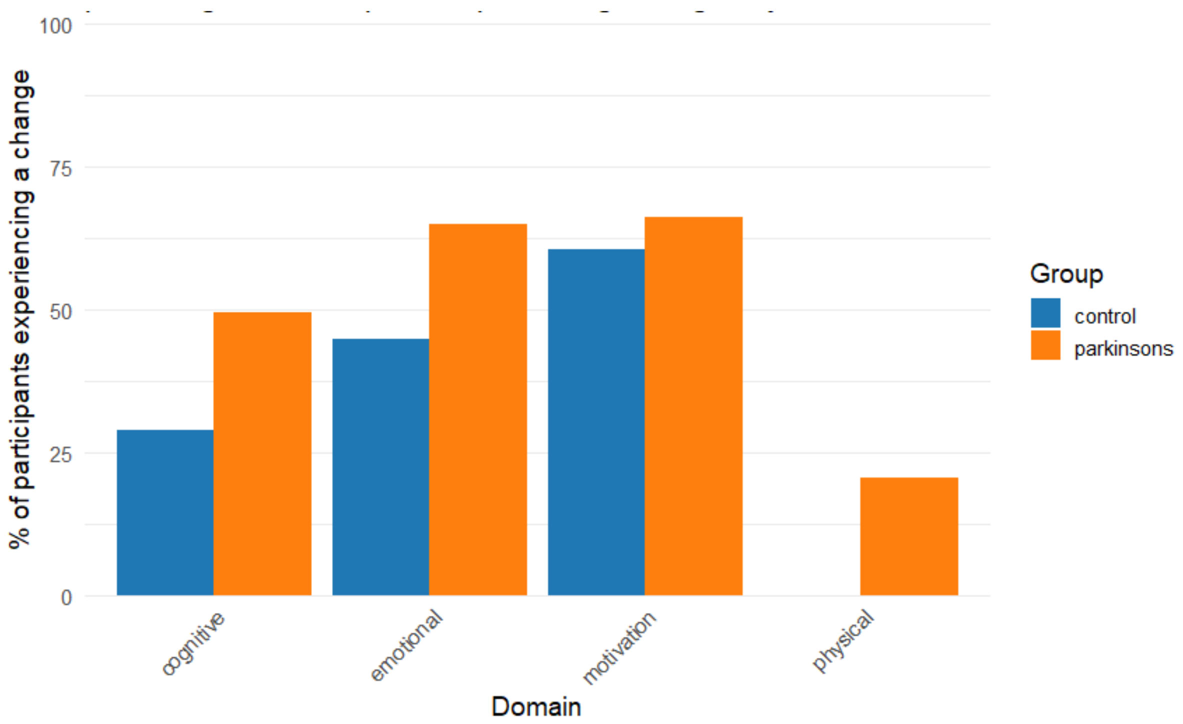

Figure S16. Percentage of participants experience changes by domain.
